# Examination of the Cell Cycle Dependence of Cytosine and Adenine Base Editors

**DOI:** 10.3389/fgeed.2022.923718

**Published:** 2022-07-14

**Authors:** Cameron A. Burnett, Ashley T. Wong, Carlos A. Vasquez, Colleen A. McHugh, Gene W. Yeo, Alexis C. Komor

**Affiliations:** ^1^ Department of Chemistry and Biochemistry, University of California, San Diego, La Jolla, CA, United States; ^2^ Institute for Genomic Medicine, University of California, San Diego, La Jolla, CA, United States; ^3^ Department of Cellular and Molecular Medicine, University of California, San Diego, La Jolla, CA, United States

**Keywords:** genome editing, DNA repair, base editing, cell cycle, genome editing and engineering

## Abstract

Base editors (BEs) are genome editing agents that install point mutations with high efficiency and specificity. Due to their reliance on uracil and inosine DNA damage intermediates (rather than double-strand DNA breaks, or DSBs), it has been hypothesized that BEs rely on more ubiquitous DNA repair pathways than DSB-reliant genome editing methods, which require processes that are only active during certain phases of the cell cycle. We report here the first systematic study of the cell cycle-dependence of base editing using cell synchronization experiments. We find that nickase-derived BEs (which introduce DNA backbone nicks opposite the uracil or inosine base) function independently of the cell cycle, while non-nicking BEs are highly dependent on S-phase (DNA synthesis phase). We found that synchronization in G1 (growth phase) during the process of cytosine base editing causes significant increases in C•G to A•T “byproduct” introduction rates, which can be leveraged to discover new strategies for precise C•G to A•T base editing. We observe that endogenous expression levels of DNA damage repair pathways are sufficient to process base editing intermediates into desired editing outcomes, and the process of base editing does not significantly perturb transcription levels. Overall, our study provides mechanistic data demonstrating the robustness of nickase-derived BEs for performing genome editing across the cell cycle.

## Introduction

**FIGURE 1:**
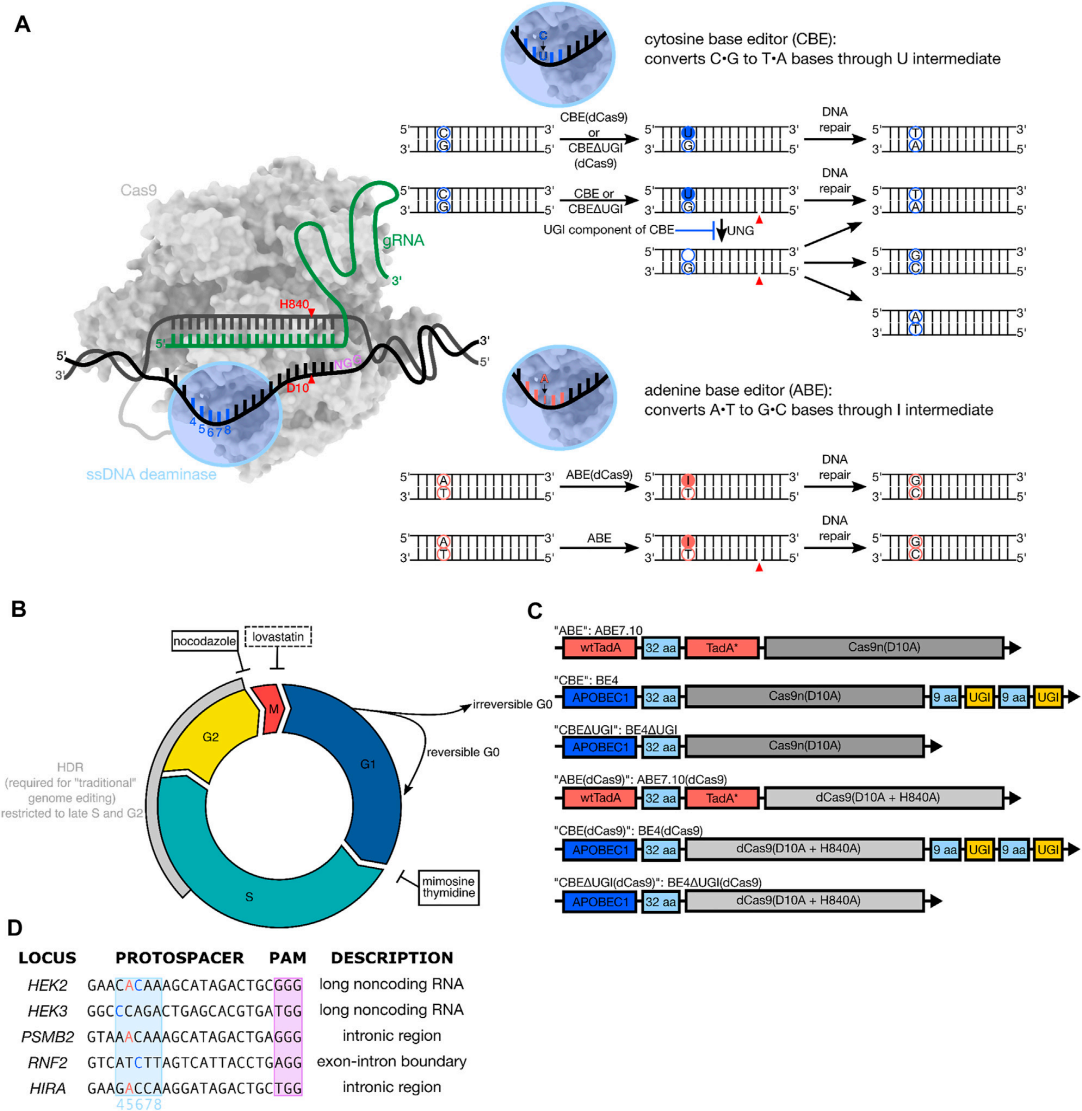
**(A)** Overview of base editing. Base editors consist of a ssDNA modifying enzyme (blue) covalently tethered to a catalytically inactive or impaired Cas9 (dCas9 or Cas9n) protein (grey). The base editor binds to a genomic locus of interest *via* protospacer adjacent motif (PAM, shown in purple) recognition and Watson-Crick-Franklin base pairing between the gRNA and the protospacer. Local denaturation of the DNA during R-loop formation exposes ∼5 nucleotides (protospacer positions 4–8) to the ssDNA modifying enzyme, which will deaminate the target base to the U or I intermediate. The modified base will get permanently converted to a canonical base pair following cellular replication or repair across the lesion. **(B)** Overview of the phases of the cell cycle. Precision genome editing using wild-type Cas9 uses homology directed repair, which is highly cell cycle dependent (grey arc) and usually outcompeted by the error-prone non-homologous end joining pathway. Base editing has been demonstrated to be effective in terminally differentiated cells, suggesting its reliance on non-cell-cycle-dependent DNA repair pathways. The chemical synchronization agents used in this study (nocodazole, mimosine, and thymidine) are indicated in black boxes with inhibitory arrows pointing to the phase of the cell cycle in which they arrest cells. Additionally, the synchronization agent lovastatin, which was used in initial synchronization experiments but not in base editing experiments, is indicated in a black dashed box. **(C)** Architecture of constructs used. All constructs employ the *Streptococcus pyogenes* (Sp) Cas9 homolog. All ABEs are single polypeptide chains consisting of three fused protein components—a wild-type *Escherichia coli* (*E. coli*) TadA (tRNA-specific adenosine deaminase), a laboratory evolved *E. coli* TadA (TadA*) that catalyzes deoxyadenosine deamination, and an impaired (Cas9n) or inactive (dCas9) Cas9. All CBEs are a single protein fusion consisting of the rat APOBEC cytidine deaminase enzyme (APOBEC1) tethered to Cas9n or dCas9, followed by two copies of uracil glycosylase inhibitor (UGI)–except in the case of the CBE∆UGI constructs. **(D)** Protospacers and PAM (purple) sequences of the genomic loci studied, with target Cs (blue) and As (salmon) within the base editing window (protospacer positions 4–8, light blue) indicated.

Base editing is a “nontraditional” genome editing method that utilizes the programmability of CRISPR-Cas9, but avoids the use of double-strand DNA breaks (DSBs) to introduce single nucleotide variants (SNVs) in the genome of live cells (Komor et al., 2016; Gaudelli et al., 2017). These tools consist of a single-stranded DNA (ssDNA)-specific deaminase enzyme fused to a catalytically inactivated or impaired Cas protein (dCas9, dCas12, or Cas9n) (Li et al., 2018). Due to the enzymes’ requirements for ssDNA, deamination of target nucleobases is confined to a small (∼5 nucleotide) window within the Cas9:gRNA:DNA R-loop (Figure 1A). Two main classes of base editors have been developed: cytosine base editors (CBEs) (Komor et al., 2016; Nishida et al., 2016), which employ cytidine deaminase enzymes to convert C•G base pairs to predominantly T•A outcomes *via* uracil-containing intermediates, and adenine base editors (ABEs), which employ evolved adenosine deaminase enzymes to convert A•T base pairs to G•C *via* inosine-containing intermediates (Gaudelli et al., 2017) (Figure 1A). BEs utilize either a completely catalytically inactive dCas9 (in which case the only DNA modification is the introduction of the modified base, Figure 1A), or a Cas9n, in which case the strand opposite the modified base is nicked to manipulate DNA repair processes to preferentially replace this strand, using the modified base as a template (Figure 1A). Notably, cytosine base editing can result in C•G to non-T•A editing outcomes at certain sites through a mechanism that is not currently well-understood but involves excision of the uracil intermediate by uracil N-glycosylase (UNG) (Komor et al., 2017). These outcomes can be suppressed by incorporating into the BE architecture a polypeptide called Uracil Glycosylase Inhibitor (UGI), which binds irreversibly to the UNG protein. In contrast, ABEs produce more precise editing outcomes, with minimal A•T to non-G•C conversions. “Traditional” genome editing by wild type (wt) CRISPR-Cas9, on the other hand, relies on the initial introduction of DSBs, followed by DNA repair manipulation to achieve precise editing outcomes (Cho et al., 2013; Cong et al., 2013; Jinek et al., 2013; Mali et al., 2013). Two main pathways compete to process Cas-mediated DSBs: non-homologous end joining (NHEJ), which introduces insertion and deletion (indel) products at the DSB site, while homology-directed repair (HDR) uses an exogenously-supplied donor DNA template to introduce precise modifications near the site of the DSB (Mao et al., 2008). A major limitation of traditional, DSB-mediated genome editing is that DSBs are typically repaired more efficiently by NHEJ than HDR. Additionally, the cell-cycle dependent expression of HDR machinery (which is mainly expressed during the DNA synthesis phase, or S phase, of the cell cycle) has limited the use of HDR-mediated precision genome editing tools to cell types which are actively proliferating (Shrivastav et al., 2008). A variety of strategies involving modulation of DSB repair factors have been developed to improve the ratio of HDR to NHEJ outcomes due to our detailed understanding of the underlying DNA repair mechanisms involved in DSB-mediated genome editing (Chu et al., 2015; Riesenberg and Maricic, 2018; Liu et al., 2019). In contrast, the DNA repair mechanisms that process base editing intermediates are not well understood.

Repair of DNA damage is mediated by several different repair pathways, which are active to varying degrees throughout different stages of the cell cycle (Branzei and Foiani, 2008). For example, the HDR machinery is primarily expressed during the late Synthesis (S) and Gap 2 (G2) phases of the cell cycle (Branzei and Foiani, 2008; Lin et al., 2014) (Figure 1B). In fact, one strategy to improve HDR to NHEJ ratios in DSB-reliant genome editing experiments involved pre-synchronizing cells prior to delivery of Cas9:sgRNA ribonucleoprotein complex and donor template to coordinate the initial “burst” of genome editing activity with S- and/or G2/M- phase (Lin et al., 2014). Base editing, in contrast, has been hypothesized to rely on the mismatch repair (MMR) and/or base excision repair (BER) pathways, which are thought to be less drastically regulated by the phases of the cell cycle than HDR. In support of this, several studies have reported successful base editing in post-mitotic, non-proliferating cell types (Yeh et al., 2018; Lim et al., 2020). However, no systematic investigation of the cell cycle-dependence of base editing has been conducted. A detailed understanding of how base editing outcomes can change with respect to the cell cycle would inform us on the cell types most amenable to efficient and precise base editing, reveal new strategies to enhance certain base editing outcomes, and provide more information about the DNA repair mechanisms by which these tools operate.

Here, we utilize chemical synchronization to arrest human embryonic kidney 293T (HEK293T) and human erythroleukemic (K562) cells at G1 or G2/M and quantify changes in base editing efficiency and precision for ABE7.10, BE4, and BE4∆UGI, as well as the corresponding constructs with dCas9 instead of Cas9n (see Figures 1A,C), hereafter referred to as ABE, CBE, CBE∆UGI, ABE (dCas9), CBE (dCas9), and CBE∆UGI (dCas9), respectively. We quantify changes in efficiency and precision at three distinct genomic loci per construct, which represent both coding and non-coding regions (Figure 1D). We observe small changes (less than 25% for CBE, and less than 45% for ABE) in overall base editing efficiencies with respect to cell cycle synchronization for the Cas9n-derived BEs (which install DNA backbone nicks in the strand opposite to the uracil or inosine intermediate), and drastic reductions (∼70% reductions for ABE and ∼80% reductions for CBE) in overall base editing efficiencies for the dCas9-derived BEs for both synchronization conditions. These data suggest that fundamentally different DNA repair mechanisms process the different intermediates into desired editing outcomes, with nicked intermediates relying on more ubiquitous pathways, and non-nicked intermediates relying heavily on S phase-dependent pathways to be processed into desired outcomes. Additionally, we observe large increases in relative C•G to A•T rates by CBE∆UGI (which lacks the UGI component of CBE and therefore allows high levels of excision of the uracil intermediate) upon synchronization in G1. This discovery can be leveraged to identify new strategies for precise C•G to A•T base editing, similarly to recent methods using CBEs for precise C•G to G•C base editing (Chen et al., 2021; Kurt et al., 2021; Zhao et al., 2021). To relate these results in the context of DNA repair pathways, we additionally perform bulk RNA expression profiling experiments and analyze expression level changes of over 20,000 RNAs during base editing, but do not observe any notable statistically significant changes. This suggests that endogenous transcription levels of DNA damage repair pathways are sufficient to process base editing intermediates into desired editing outcomes, and the process of base editing does not significantly perturb steady state mRNA levels.

## Results

### Timeline of Base Editing

To determine the optimal experimental conditions for combining cell synchronization with quantification of base editing outcomes, we first conducted a time course experiment to observe the kinetics of editing by CBE, CBE∆UGI, and ABE with established gRNAs (Supplementary Figure S1). We transfected HEK293T cells with plasmids encoding BE and gRNA and extracted genomic DNA (gDNA) at 12, 18, 24, 36, 48, 72, and 96 h post-transfection. Genomic loci of interest were amplified, subjected to high throughput sequencing (HTS), and analyzed for genome editing efficiencies using CRISPResso2 (Clement et al., 2019). We observed a gradual, consistent increase in editing efficiency over time by ABE (Supplementary Figure S1A) throughout the course of the entire experiment. In contrast, editing by CBE appeared to peak at 36 h, followed by a slight decrease at 48 h which then recovered and increased through the end of the experiment (Supplementary Figure S1B). Editing by CBEΔUGI peaked at 48 h and then decreased through the end of the experiment (Supplementary Figure S1C). When each datapoint is normalized to the highest editing observed for each locus and then averaged across all three loci for each construct, 24 ± 10%, 54 ± 7%, and 40 ± 12% of overall editing is observed within 18 h of transfection by ABE, CBE, and CBEΔUGI, respectively (mean ± SD for *n* = 3 biological replicates per site, averaged over three different sites). These differences in the timelines of editing between ABE and CBE could be due to differences in protein expression/stability, inherent differences in how quickly the two intermediates are introduced by their respective enzymes, or due to differences in the timing of how the two intermediates are processed by the cell.

### Chemical Inhibitors Arrest Cells After 12 Hours of Treatment

Cells can be arrested at specific phases of the cell cycle using chemical inhibitors such as lovastatin (G1 arrest), mimosine (G1/S boundary, prior to replication), thymidine (G1/S boundary, prior to replication) and nocodazole (G2/M) (Shrivastav et al., 2008) (Figure 1B). To determine the time frame of synchronization for our experiments, we treated HEK293T and K562 cells with each of these chemical inhibitors and monitored synchronization of the cells at 6, 12, and 18 h by examining the fluorescence of fixed and propidium iodide-stained cells. We used flow cytometry to visualize DNA content and quantified the fraction of cells in the population that are in G1, S, and G2/M phases following chemical synchronization.

Unsynchronized HEK293T cells exhibit on average 43 ± 1% of cells in G1, 42 ± 1% of cells in S, and 15 ± 0.2% of cells in G2/M (mean ± SD for *n* = 3 biological replicates, Supplementary Figure S2). We observed a modest increase in synchronization of cells after 6 h of nocodazole treatment (28 ± 3% of cells in G1, 26 ± 1% of cells in S, and 46 ± 1.5% of cells in G2/M, Supplementary Figure S2), which drastically increased by 12 h post-treatment to 4 ± 4% of cells in G1, 14 ± 1.5% of cells in S, and 82 ± 4% of cells in G2/M (Supplementary Figure S2). Moderate synchronization was also observed after 6 h of treatment with thymidine (60 ± 6% of cells in G1, 40 ± 6% of cells in S, and 0.1 ± 0.1% of cells in G2/M, Supplementary Figure S2), but 12 h of treatment was able to arrest cells at G1 and early S phase, with 80 ± 6% of cells in G1, 19 ± 6% of cells in S, and 0.7 ± 0.5% of cells in G2/M (Supplementary Figure S2). Mimosine treatment also arrested cells at the G1/S border after 12 h, with 59 ± 1% of cells in G1, 37 ± 1% of cells in S, and 0.5 ± 0.9% of cells in G2/M. Analogous treatment of K562 cells with these three chemicals produced similar results (Supplementary Figure S2). In contrast, lovastatin had no effect on either HEK293T or K562 cell cycle distribution with up to 18 h of treatment (Supplementary Figure S2). Cells held synchronized for more than 48 h were determined to be non-viable by Trypan Blue staining (Supplementary Figure S3).

### Delaying Synchronization Is Required to Maintain Equal Base Editors Expression Levels

**FIGURE 2:**
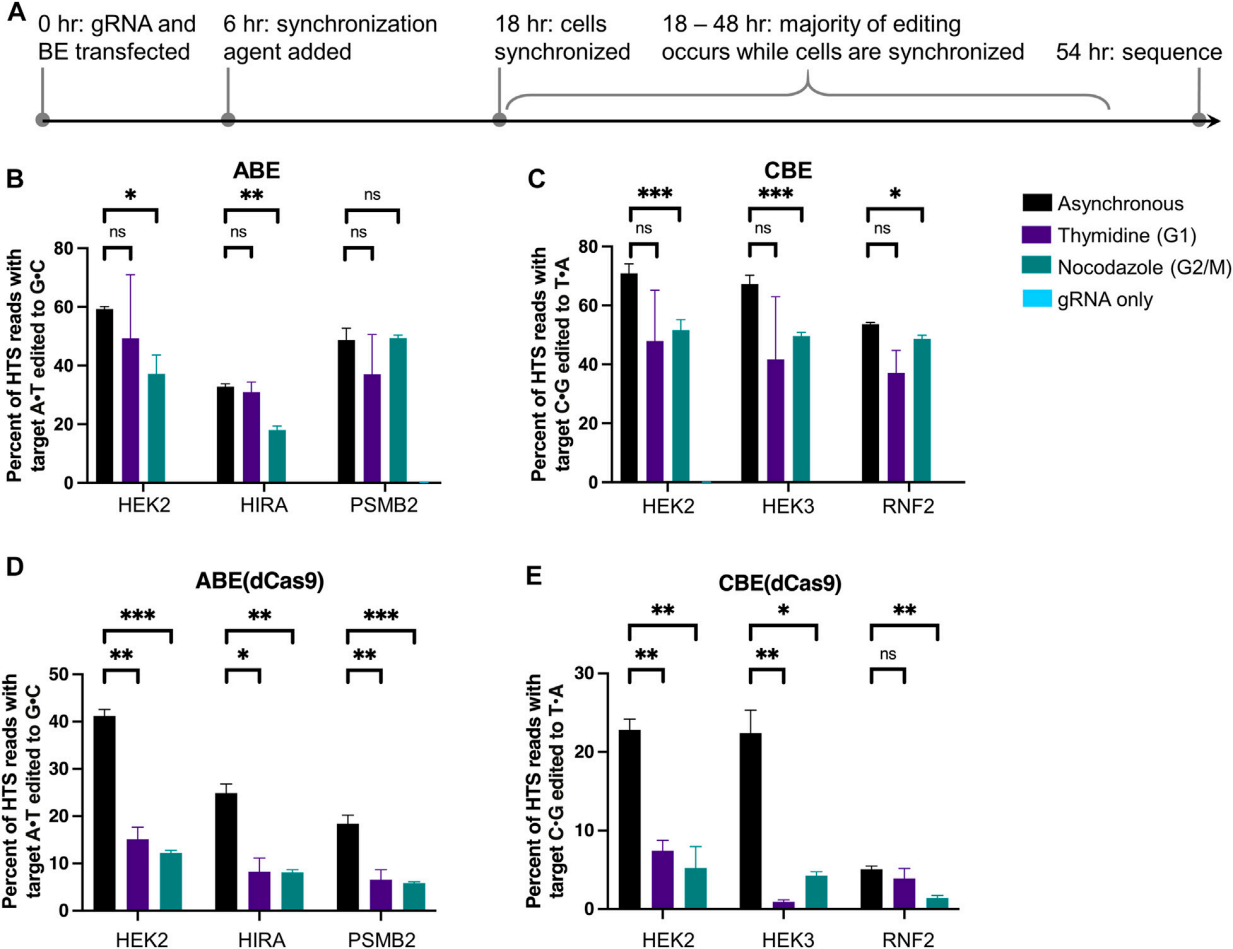
Cell cycle synchronization effects on base editing efficiencies and precision of ABE, ABE (dCas9), CBE, and CBE (dCas9) in HEK293T cells. **(A)** Cells were transfected with BE plus gRNA (protospacer sequences indicated in Figure 1), synchronization agents were added 6 h post-transfection (thymidine for G1 synchronization or nocodazole for G2/M synchronization), and cells were lysed at 54 h. As a negative control, cells were transfected with gRNA plasmid only (gRNA only sample). The genomic DNA was extracted, and target loci were amplified *via* PCR and subjected to high-throughput sequencing (HTS). Genome editing efficiencies [percent of total HTS reads with the target A•T base converted to G•C for ABE and ABE (dCas9), or percent of total HTS reads with the target C•G base converted to T•A for CBE and CBE (dCas9)] were quantified with CRISPResso2. Base editing efficiencies by ABE **(B)**, CBE **(C)**, ABE (dCas9) **(D)**, and CBE (dCas9) **(E)** are plotted. Values and error bars reflect the means and SD of three independent biological replicates performed on different days. Asterisks reflect *p* value calculations of unpaired *t* test, one tailed (ns indicates not significant, *p < 0.05, **p < 0.01, ***p < 0.001).

We performed initial experiments by slightly modifying established protocols (Jackman and O’Connor, 1998; Lin et al., 2014). Synchronization agent was added first to HEK293T cells, followed by transfection of BE and gRNA plasmids after 17 h. At 24 h post-transfection, flow cytometry was used to measure and compare green fluorescent protein (GFP) expression levels of the synchronized samples to that of the unsynchronized samples (using BE-P2A-GFP constructs, in which BE and GFP are transcribed together but translated into separate proteins) (Liu et al., 2017). We observed drastic reductions in BE expression levels when cells were pre-synchronized with nocodazole or mimosine, most likely due to a reduction in either transfection efficiency or protein translation rates caused by synchronization (a decrease from an average of 60 ± 5% of cells exhibiting GFP fluorescence for asynchronous cells to 32 ± 1% for G1 arrested cells by mimosine and 24 ± 2% for G2/M arrested cells by nocodazole, with thymidine within error at 57 ± 3% GFP, mean ± SD for *n* = 3 biological replicates, Supplementary Figure S3). Adding synchronization agents at the time of transfection improved the percentage of GFP positive cells to 49 ± 5% for mimosine (G1 arrested), 43 ± 5% for nocodazole treated cells (G2/M arrested), and 57 ± 2% for thymidine treated cells (G1 arrested) at 24 h post-transfection. However, delaying the addition of synchronization agents until 6 h after transfection was determined to be the best balance between preserving GFP (and therefore BE) expression levels, while ensuring the majority of base editing activity (as determined by our time-course experiments) occurred when cells were synchronized. Specifically, 62 ± 3% of mimosine treated cells (G1 arrested, no statistically significant difference), 52 ± 5% of nocodazole treated cells (G2/M arrested, representing a 16 ± 8% decrease in GFP fluorescence compared to asynchronous cells), and 57 ± 2% of thymidine treated cells (G1 arrested, no statistically significant difference) exhibited GFP fluorescence at 24 h post-transfection. We chose to move forward using thymidine as our G1 synchronization agent due to its more complete synchronization of cells in G1. By delaying addition of synchronization agents until 6 h after transfection, cells are fully synchronized by 18 h post-transfection, allowing us to observe changes in editing efficiency and precision that occur between 18 and 54 h post-transfection (indicated in Supplementary Figure S1D with the dotted box and Figure 2A) due to synchronization.

### Synchronization Effects on Adenine Base Editors

Informed by our previous experiments, HEK293T cells were transfected with ABE or ABE (dCas9) and gRNA, then treated with synchronizing agents for 48 h beginning 6 h post-transfection. Cells were lysed, gDNA was extracted, and genomic loci of interest were amplified, subjected to HTS, and analyzed for genome editing efficiencies using CRISPResso2 (Figure 2). We confirmed that cells remained synchronized following transfection at 18 and 54 h in a 48-well plate format (Supplementary Figure S4). These two editors differ in that ABE (dCas9) would produce an intermediate lacking a nick on the strand across from the inosine. While dCas9-derived BEs are in general less commonly used than their Cas9n counterparts due to their reduced overall efficiencies, we were interested in observing the impact that nicking of the unedited strand has on the cell cycle dependence of base editing. Notably, we observed no statistically significant changes in A•T to G•C editing efficiencies by ABE at any of the three sites (the *HEK2*, *HIRA*, and *PSMB2* loci) upon synchronization in G1 using thymidine relative to asynchronous populations (*p* > 0.05 two-tailed Student’s *t*-test, Figure 2B). When cells were synchronized in G2/M using nocodazole, we observed a 37 ± 11% decrease in A•T to G•C editing efficiency at the *HEK2* site, a 45 ± 5% decrease at the *HIRA* site, and no reduction at the *PSMB2* site (Figure 2B). We repeated these experiments in K562 cells and observed the same overall trends, demonstrating the generality of these data (Supplementary Figure S5A). In direct contrast, A•T to G•C editing efficiencies by ABE (dCas9), significantly decreased in HEK293T cells at all three sites with both synchronization conditions. When synchronized in G1 by thymidine, A•T to G•C editing decreased by 63 ± 7% at the *HEK2* site, 67 ± 15% at the *HIRA* site, and 64 ± 16% at the *PSMB2* site (mean ± SD for *n* = 3 biological replicates). When synchronized in G2/M by nocodazole, A•T to G•C editing decreased by 70 ± 4% at the *HEK2* site, 68 ± 9% at the *HIRA* site, and 68 ± 12% at the *PSMB2* site (Figure 2D). These experiments were also repeated in K562 cells and we observed the same drastic decreases in A•T to G•C editing efficiencies by ABE (dCas9) at two of the three genomic loci (Supplementary Figure S6A). These data indicate clear differences in the mechanisms by which these two editors’ intermediates are processed. Additionally, the nickase-derived ABE (which is more commonly used than the dCas9-derived ABE due to its higher efficiency) displays minimal cell cycle dependence, in direct contrast to DSB-reliant genome editing methods.

### Synchronization Effects on Cytosine Base Editor Editing Efficiencies

We repeated these experiments with CBE and CBE (dCas9) as well. Editing by CBE produced mainly C•G to T•A editing products (product purity, the percent of edited reads in which the target C•G is edited to a T•A, was >80% at all three sites, Supplementary Figure S7A), thus we analyzed only these outcomes. Upon G1 synchronization using thymidine, average C•G to T•A editing efficiencies by CBE decreased slightly at all three sites (Figure 2C), but these decreases were not statistically significant (*p* > 0.05 two-tailed Student’s *t*-test). Upon G2/M synchronization using nocodazole, editing decreased by 27 ± 7% at the *HEK2* site, 26 ± 5% at the *HEK3* site, and 5 ± 3% at the *RNF2* site (Figure 2C, mean ± SD for *n* = 3 biological replicates). However, these decreases could be attributed to the decrease in BE expression levels due to synchronization as described earlier (16 ± 8% decrease in GFP expression levels after synchronization with nocodazole). Analogous experiments in K562 cells again yielded comparable results (Supplementary Figure S5B). C•G to T•A editing efficiencies by CBE (dCas9) drastically decreased at two of the three sites with both synchronization conditions (Figure 2E); when synchronized in G1 by thymidine editing decreased by 67 ± 9% at the *HEK2* site and 96 ± 18% at the *HEK3* site, and when synchronized in G2/M by nocodazole editing decreased by 77 ± 14% at the *HEK2* site and 81 ± 17% at the *HEK3* site. C•G to T•A editing efficiencies by CBE (dCas9) at the *RNF2* locus in asynchronous cells were quite low (5 ± 0.4%), but we observed a 72 ± 12% decrease upon synchronization in G2/M by nocodazole and no statistically significant decrease upon synchronization in G1 by thymidine. We repeated these experiments in K562 cells and again observed the same overall trends (Supplementary Figure S6B). Taken together with the ABE synchronization results, these data suggest that both types of dCas9-derived BEs rely heavily on S-phase-dependent pathways to incorporate their respective point mutations, while Cas9n-derived BEs are much less cell cycle dependent in comparison.

### Synchronization Effects on CBEΔUGI Product Purity and Editing Efficiency

**FIGURE 3:**
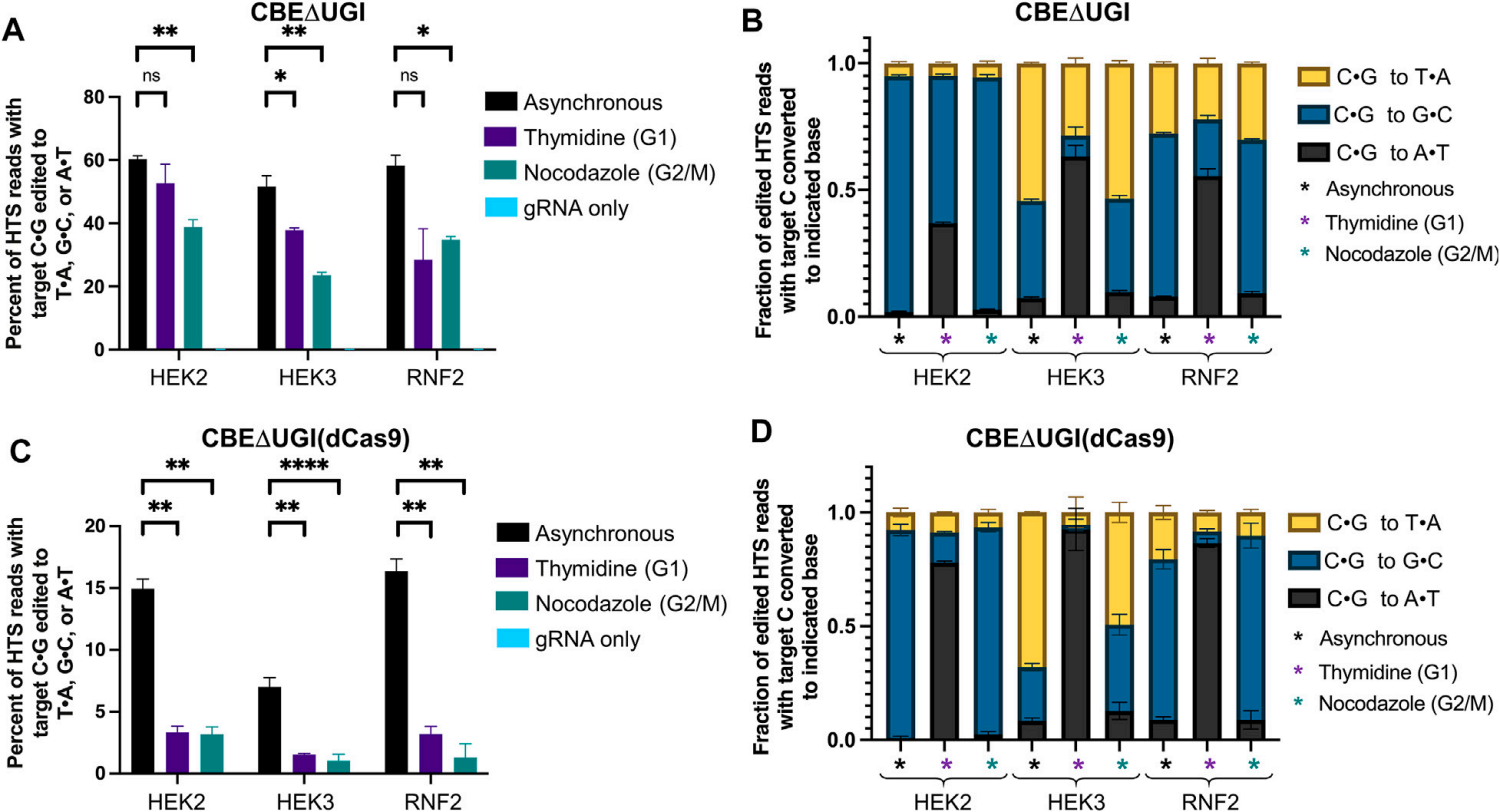
Cell cycle synchronization effects on base editing efficiencies of CBEΔUGI and CBE∆UGI (dCas9) in HEK293T cells. Cells were transfected with CBEΔUGI or CBEΔUGI (dCas9) plus gRNA (protospacer sequences indicated in Figure 1), synchronization agents were added 6 h post-transfection (thymidine for G1 synchronization or nocodazole for G2/M synchronization), and cells were lysed at 54 h. The genomic DNA was extracted and target loci were amplified *via* PCR and subjected to HTS. Genome editing efficiencies (percent of total HTS reads with the target C•G base converted to T•A, G•C, or A•T) were quantified with CRISPResso2. Base editing efficiencies by CBEΔUGI **(A)** and CBEΔUGI (dCas9) **(C)** upon synchronization are plotted. **(B,D)** The product distribution, defined as the relative portion of edited sequencing reads (reads in which the target C•G is mutated to T•A, A•T, or G•C) that have been edited to each of the indicated outcomes, is plotted for CBEΔUGI **(B)** and CBEΔUGI (dCas9) **(D)**. Values and error bars reflect the means and SD of three independent biological replicates performed on different days. Asterisks reflect *p* value calculations of unpaired *t* test, one tailed (ns indicates not significant, *p < 0.05, **p < 0.01, ***p < 0.001, p****<0.0001).

Finally, we repeated these experiments with CBEΔUGI and CBE (dCas9)ΔUGI. Because these constructs lack the uracil glycosylase inhibitor component, excision of the uracil intermediate is quite efficient, resulting in high levels of C•G to non-T•A outcomes when using these constructs. This in turn allowed us to observe changes in base editing precision with respect to cell cycle synchronization. We analyzed overall editing efficiencies (percent of HTS reads with the target C•G edited to T•A, G•C, or A•T) and product distributions (the relative portion of edited sequencing reads in which the target C•G is edited to T•A, G•C, or A•T) of each of the samples treated with CBE∆UGI (Figures 3A,B). Consistent with previous studies (Komor et al., 2017), we observed high rates of C•G to G•C editing in asynchronous cells, particularly at the *HEK2* site, allowing for observation of changes in these relative efficiencies upon synchronization. To our surprise, cells synchronized in G1 using thymidine exhibited a significant increase in the relative fraction of edited reads with C•G to A•T mutations (which increased 19 ± 1.9 -fold at the *HEK2* site, 9 ± 0.8 -fold at the *HEK3* site, and 7 ± 0.4 -fold at the *RNF2* site, Figure 3B), with an accompanying decrease in relative C•G to G•C outcomes (which decreased by 1.6 ± 0.0 -fold at the *HEK2* site, 5 ± 0.10 -fold at the *HEK3* site, and 2.9 ± 0.0 -fold at the *RNF2* site, Figure 3B), and a minimal change in the relative fraction C•G to T•A edits (which was equivalent at the *HEK2* site, decreased by 1.9 ± 0.2 -fold at the *HEK3* site, and was equivalent at the *RNF2* site, Figure 3B). In addition to relative amounts, absolute C•G to A•T point mutation efficiencies also increased upon G1 arrest at all three sites (Supplementary Figures S7C–E; absolute C•G to A•T efficiencies increased 17 ± 3-fold at the *HEK2* site, 6 ± 2-fold at the *HEK3* site, and 3 ± 1 -fold at the *RNF2* site). To further confirm this phenomenon of drastic increases in C•G to A•T editing activity upon G1 synchronization, these experiments were repeated at three additional sites. At all three sites tested, there was a substantial increase in both the absolute and fractional C•G to A•T editing (Supplementary Figures S8A–D). These results indicate that the use of G1 synchronization agents can be used as a viable option for targeted C•G to A•T base editing. Upon G2/M synchronization, absolute C•G to A•T point mutation efficiencies were within error of asynchronous cells at the *HEK2* site, and slightly decreased at the *HEK3* and *RNF2* sites (1.7 ± 0.5 and 1.4 ± 0.3-fold reductions, respectively, Supplementary Figures S7C–E). Absolute C•G to G•C introduction efficiencies were highest in asynchronous cells, followed by G2/M synchronized (decreased by 36 ± 4% at the *HEK2* site, 56 ± 6% at the *HEK3* site, and 44 ± 7% at the *RNF2* site compared to asynchronous cells, Supplementary Figures S7C–E), and lowest in G1 synchronized cells (decreased by 46 ± 6% at the *HEK2* site, 84 ± 10% at the *HEK3* site, and 83 ± 10% at the *RNF2* site compared to asynchronous cells). These results were also observed in our K562 experiments (Supplementary Figures S5C–E), although the relative changes were less drastic, potentially due to the lower overall levels of editing in this cell line. To control for changes that may be due to the chemical synchronization agent, we repeated these experiments using mimosine to synchronize the cells in G1 and observed the same increase in relative C•G to A•T rates (Supplementary Figure S9). We will note that we observed drastic decreases in overall editing efficiencies by CBE∆UGI upon G1 synchronization with mimosine that were not observed upon G1 synchronization with thymidine. We attribute this to the differences in synchronization mechanisms by the two compounds; mimosine functions *via* chelation of iron, which many DNA repair proteins require for proper folding and function.

Overall editing percentages by CBE∆UGI (dCas9) decreased dramatically at all three sites with both synchronization conditions, consistent with the ABE (dCas9) and CBE (dCas9) results (editing decreased by 78 ± 7% at the *HEK2* site, 78 ± 13% at the *HEK3* site, and 80 ± 9% at the *RNF2* site for cells synchronized in G1 by thymidine, and 79 ± 8% at the *HEK2* site, 85 ± 16% at the *HEK3* site, and 92 ± 11% at the *RNF2* site for cells synchronized in G2/M by nocodazole, Figure 3C). Even though overall editing efficiencies were below 5% at all three sites upon synchronization, we still observed the same trends in C•G to non-T•A editing outcomes upon synchronization (statistically significant increases in C•G to A•T introduction efficiencies upon G1 synchronization, and statistically significant decreases in C•G to G•C editing efficiencies upon G1 and G2/M synchronization, with higher absolute C•G to G•C editing efficiencies in G2/M synchronized cells compared to G1 synchronized cells, Figure 3D and Supplementary Figure S10). Again, these experiments were repeated in K562 cells and comparable results were obtained (Supplementary Figure S6C).

### Analysis of Changes in Indel Sequences and Introduction Efficiencies by Cas9n-Derived Cytosine Base Editors Following Synchronization

**FIGURE 4:**
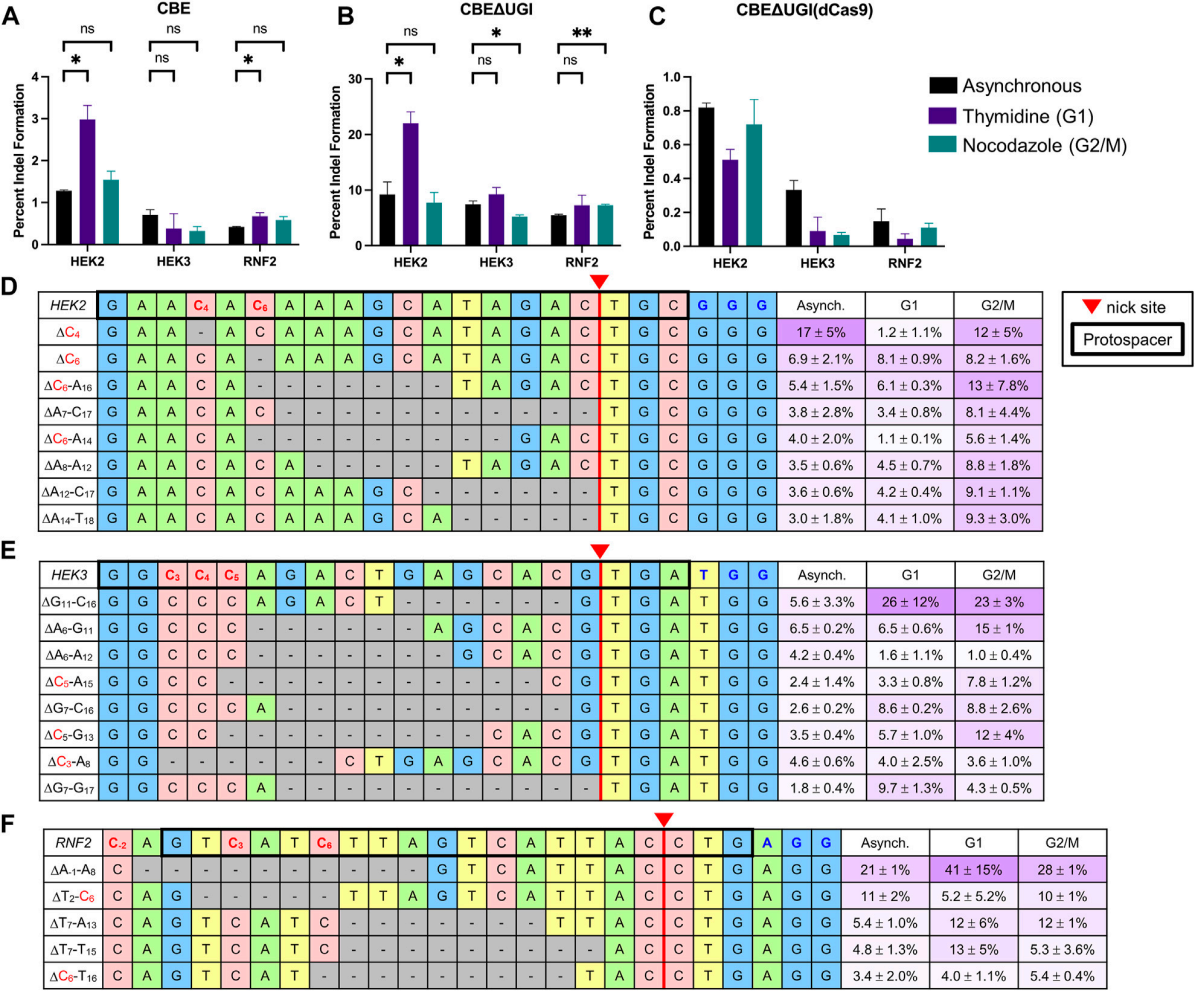
Indel analysis of CBEs in HEK293T cells. HEK293T cells were transfected with CBE **(A)**, CBEΔUGI **(B)**, or CBEΔUGI (dCas9) **(C)** plus gRNA (protospacer sequences indicated in Figure 1), synchronization agents were added 6 h post-transfection (thymidine for G1 synchronization or nocodazole for G2/M synchronization), and cells were lysed at 54 h. The genomic DNA was extracted and target loci were amplified *via* PCR and subjected to HTS. Total indel introduction efficiencies for CBE **(A)**, CBEΔUGI **(B)**, and CBEΔUGI (dCas9) **(C)** were calculated as the percent of reads with insertions or deletions (determined *via* CRISPResso analysis) divided by the total number of HTS reads sequenced. **(A–C)** Effects of synchronization in G1 or G2/M on indel introduction efficiencies. **(D–F)** The most common (defined as sequences that comprise greater than 0.1% of total reads in at least two out of three of the asynchronous, G1-synchronized, and G2/M-synchronized samples) indel sequences are shown with respect to the protospacer (bold outline), potential edited cytosines (indicated in red), nick site (red triangle), and PAM (indicated in blue) for the *HEK2* **(C)**, *HEK3* **(D)**, and *RNF2* **(E)** sites. The relative portion of total indel reads with each specific indel sequence is listed on the left in purple with respect to synchronization condition (note these are not absolute indel introduction efficiencies). Values and error bars reflect the means and SD of three independent biological replicates performed on different days. Asterisks reflect *p* value calculations of unpaired *t* test, one tailed (ns indicates not significant, *p < 0.05, **p < 0.01, ***p < 0.001).

We additionally analyzed indel formation by all BEs in asynchronous and synchronized cells. Indel rates by ABE and dCas9-derived BEs were generally below 1% for all three sites at all conditions, consistent with previous reports (Figures 4A,C; Supplementary Figures S11A–D) (Rees and Liu, 2018). Indel rates by CBE were higher than those by ABE, but still generally less than 1% except at the HEK2 site (Supplementary Figures S11A,C). However, indel rates by CBE∆UGI were on average 10 ± 3-fold higher than those by CBE at the exact same sites and under the same conditions, suggesting the involvement of UNG in CBE-induced indels (Figures 4A,B). Additionally, indel rates by CBE∆UGI were between 11- and 100-fold higher than those by CBE∆UGI (dCas9) at the exact same sites and under the same conditions (Figures 4B,C), suggesting the involvement of nicking of the unedited strand in CBE-induced indels as well. Due to the elevated rates of indel formation by CBE∆UGI, we focused our additional analyses on indels introduced by this construct.

We observed no consistent changes in absolute indel rates by CBE∆UGI upon synchronization with either agent (upon synchronization in G1 by thymidine, absolute indel rates by CBE∆UGI increased 2.4 ± 0.5-fold at the *HEK2* site, but did not change at the *HEK3* site or *RNF2* site, and upon synchronization in G2/M by nocodazole, absolute indel rates by CBE∆UGI were equivalent at the *HEK2* site, reduced by 1.4 ± 0.1 -fold at the *HEK3* site, and increased by 1.4 ± 0.1 -fold at the *RNF2* site, Figure 4B). This may be indicative of differences in DNA repair of non-coding versus coding regions of the genome, particularly with respect to their accessibility to glycosylases. We analyzed individual indel sequences and found that, among the most common sequences (those that represent >0.1% of total reads in two out of three of the asynchronous, G1-synchronized, and G2/M synchronized samples, displayed in Figures 4D–F), deletion sequences were confined to the region between the deaminated target cytosine(s) and the location of the Cas9n-induced nick at all three sites. We observed no insertion sequences among the most common indel sequences. This is in direct contrast to DSB-mediated indels, which are centered around the Cas9 cut site. Interestingly, upon synchronization in either G1 or G2/M, the relative amounts of each indel sequence changed drastically (Figures 4D–F), indicating that certain indel sequences are preferentially produced during different phases of the cell cycle. Taken together, these observations suggest an inherently different mechanism of indel introduction by CBEs compared to DSB-reliant technologies.

We additionally treated HEK293T cells with BEs optimized for reduced RNA off-target activity (Kim et al., 2017; Zhou et al., 2019) (BE4-W90Y-R126E, BE4-W90Y-R126E-ΔUGI, and ABE7.10-F148A, referred to as CBE-YE1, CBEΔUGI-YE1, and ABE-F148, respectively, see Supplementary Figure S11E) for RNA sequencing experiments (see next results section) as well as their catalytically inactivated deaminase counterparts [referred to as CBE-E63A-YE1 (Doman et al., 2020), CBEΔUGI-E63A-YE1, and ABE-E59A-F148A (Kim et al., 2006), respectively, see Supplementary Figure S11E] and analyzed their rates of indel formation when targeting the *HEK2* site. We again observed a 12 ± 3-fold increase in indel rates in CBE∆UGI-YE1 treated cells compared to CBE-YE1 treated cells (Supplementary Figures S11G–H). When comparing each catalytically active CBE variant to its respective catalytically inactive counterpart, we observed a 3 ± 1.8-fold decrease in indel introduction efficiency for CBE-YE1 (Supplementary Figure S11G), and a 21 ± 5-fold decrease in indel introduction efficiency for CBEΔUGI-YE1 (Supplementary Figure S11H), suggesting that nicking alone is insufficient for indel formation, but requires uracil introduction as well. We were quite surprised to see that indel introduction efficiencies by ABE increased by 7 ± 1-fold upon catalytically inactivating the deaminase (Supplementary Figure S11F). These data suggest ABE-mediated indel introduction may be through an inosine-independent mechanism, although additional datapoints are needed to confirm this.

### Transcriptional Dynamics Due to Base Editing

**FIGURE 5:**
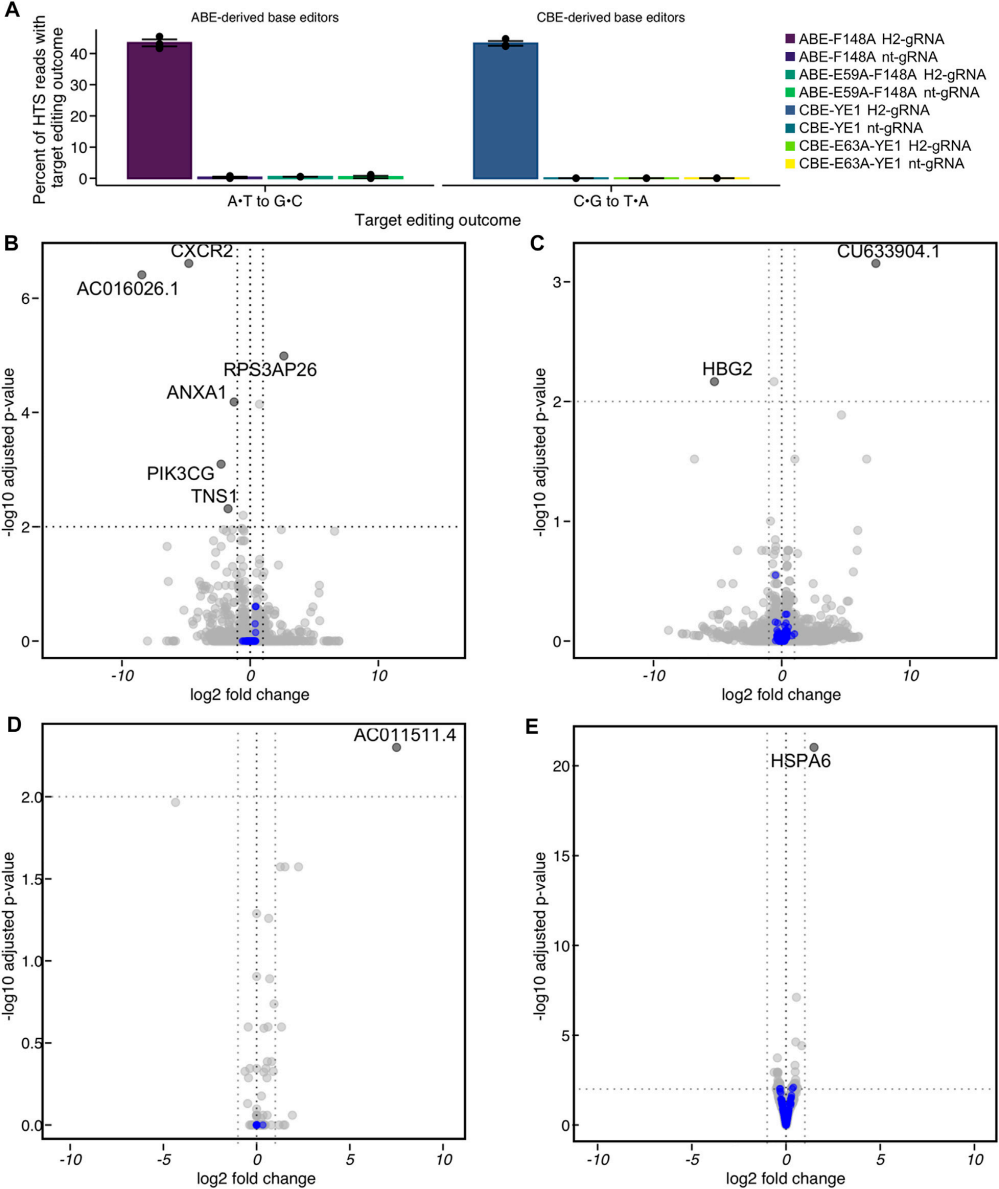
Differential expression analysis of HEK293T cells undergoing base editing at the *HEK2* (H2) genomic locus by RNA off-target optimized constructs. **(A)** HEK293T cells were transfected with the reduced-RNA editing variants ABE-F148A or CBE-YE1, or their catalytically inactivated deaminase counterparts ABE-E59A-F148A or CBE-E63A-YE1, CBE∆UGI-E63A-YE1 and either a HEK2-targeting gRNA (H2-gRNA) or a non-targeting gRNA (nt-gRNA). At 48 h post-transfection, cells were lysed, the genomic DNA was extracted, and target loci were amplified *via* PCR and subjected to HTS. Genome editing efficiencies (percent of total HTS reads with the target A•T base converted to G•C for ABE variants, or percent of total HTS reads with the target C•G base converted to T•A for CBE variants) were quantified with CRISPResso2. Shown are overall base editing efficiencies for all samples. **(B–E)** HEK293T cells were treated identically, but at 48 h post-transfection, the RNA was extracted. The coding transcriptome of the RNA libraries was enriched and subjected to HTS. **(B)** Differential expression of CBE-YE1 H2-gRNA vs. CBE-YE1 nt-gRNA. **(C)** Differential expression of CBE-YE1 H2-gRNA vs. CBE-E63A-YE1 H2-gRNA. **(D)** Differential expression of ABE-F148A H2-gRNA vs. ABE-F148A nt-gRNA. **(E)** Differential expression of ABE-F148A H2-gRNA vs. ABE-E59A-F148A H2-gRNA. Statistically significantly differentially expressed genes are labeled with their gene names, and DNA damage repair genes are colored in blue. Statistically significantly differentially expressed genes were defined as genes where the absolute value of the log2 (fold change) > 1 and the adjusted *p*-value ≤ 0.01.

To determine the transcriptomic landscape of protein-coding and non-coding RNA regulated by the process of incorporating SNVs through base editing, we performed bulk RNA sequencing (RNAseq) of HEK293T cells during the process of base editing. If endogenous levels of DNA repair proteins are not sufficient to convert BE intermediates into their respective outcomes, the corresponding RNAs may become upregulated during the process of base editing. As mentioned previously, we used nCas9-derived ABE and CBE variants with minimized off-target RNA editing activity (CBE-YE1 and ABE-F148A, shown in Supplementary Figure S11E) to avoid any RNA mutation-induced changes in RNA transcript levels. The CBE-YE1 variant also has been shown to induce significantly reduced gRNA-independent off-target DNA editing (Doman et al., 2020). Taken together, expression of these constructs would result in uracil or inosine introduction only at the on-target, or a low number of gRNA-dependent off-target, site(s) in genomic DNA. We also used the catalytically inactive deaminase versions of CBE-YE1 and ABE-F148A (CBE- E63A-YE1 and ABE-E59A-F148A, shown in Supplementary Figure S11E) as important control samples. HEK293T cells were transfected with CBE-YE1, ABE-F148A, CBE-E63A-YE1, or ABE-E59A-F148A, and either a gRNA targeting the HEK2 locus (H2-gRNA) or a non-targeting gRNA (nt-gRNA). Editing of their respective target bases within the HEK2 protospacer was quantified *via* HTS 48 h post-transfection: overall base editing efficiencies at this time were 43 ± 1% for CBE-YE1 plus H2-gRNA and 43 ± 2% for ABE-F148A plus H2-gRNA (Figure 5A), indicating that the cells were in the process of converting uracil and inosine intermediates into desired editing outcomes (mean ± SD for *n* = 3 biological replicates). All cells treated with nt-gRNA or catalytically inactive deaminase editors displayed editing efficiencies at levels of non-treated controls at the HEK2 locus (Figure 5A). Additionally, propidium iodine staining of cells at 48 h post-transfection showed cells were healthy and viable (Supplementary Figure S12A), and flow cytometry analyses showed transfection efficiencies were ≥60% (Supplementary Figure S12B).

In parallel, triplicate samples of each condition were lysed 48 h post-transfection and RNA was extracted. The coding transcriptome of the RNA libraries was enriched and subjected to HTS. We compared the transcriptome of cells treated with active BE and either H2-gRNA or nt-gRNA for both CBE-YE1 and ABE-F148A and performed differential expression analyses based on the negative binomial distribution using the *DESeq2* (Love et al., 2014) package. Differences in transcription levels between these samples could be caused by R-loop formation, DNA nicking, and/or uracil or inosine introduction at the on-target locus. We observed one statistically significant large-scale transcriptomic change occur during the process of adenine base editing (out of >23,000 sequenced RNAs, Figure 5D and Supplementary Figure S13), suggesting steady state mRNA levels are sufficient to process adenine base editing intermediates into the desired outcome, and the process of adenine base editing does not perturb the transcriptome [absolute value of the log2 fold change >1 and Wald test-attained and Benjamini-Hochberg corrected adjusted *p*-value (false discovery rate, FDR) ≤0.01]. We identified very few RNA that were differentially expressed (*n* = 1 upregulated, *n* = 5 downregulated, out of >20,000 sequenced RNAs, Figure 5B and Supplementary Figure S13) as a result of cytosine base editing, suggesting that the mechanisms by which adenine and cytosine base editing intermediates are processed may be inherently different. A gene ontology analysis of these data using *fgsea* (a package for fast pre-ranked gene set enrichment analysis) showed that these RNA were not ontologically related. Therefore, we performed a custom gene set enrichment analysis to identify if gene sets of DNA repair pathways were enriched or depleted. We compiled a list of ∼220 DNA repair genes and categorized them according to their DNA repair pathways (Milanowska et al., 2011; Olivieri et al., 2020) (Supplementary Table S1). We found that no DNA repair pathways were significantly enriched or depleted in either CBE or ABE analysis (Figure 5 blue dots).

We then performed differential expression analyses between samples treated with active BE and H2-gRNA and those treated with inactive BE and H2-gRNA for both CBE-YE1 and ABE-F148A. Expression of inactive BE plus H2-gRNA would result in R-loop formation and DNA nicking at the HEK2 locus, with no introduction of uracil or inosine. Differences in transcription levels between these samples would therefore solely be due to uracil or inosine introduction and processing. We again observed one large-scale transcriptomic change occur in the ABE samples (out of >24,000 sequenced RNAs, Figure 5E and Supplementary Figure S13), and two RNAs that were differentially expressed (out of ∼20,000 sequenced RNAs, Figure 5C and Supplementary Figure S13) in the CBE samples. Again, a gene ontology analysis showed that these RNA were not ontologically related, and a custom gene set enrichment analysis of the various DNA repair pathways revealed that none were significantly enriched or depleted (Figure 5, blue dots). A full list of all genes found to be up- or down-regulated in each comparison is listed in Supplementary Figure S13.

### Summary of Key Results

In short, we report four key findings in this work. First, we established that the mechanisms by which Cas9n-derived BEs (which function by nicking the DNA strand opposite of the modified base) function are distinct from those by which dCas9-derived BEs function. Second, we found that Cas9n-derived BEs function fairly independently of the cell cycle, while dCas9-derived BEs are highly dependent on S-phase. This observation has large implications for researchers performing genome editing experiments in non-dividing cells. Third, we found that rates of C•G to A•T base editing by CBEs are significantly increased during G1 phase. Finally, our bulk mRNAseq data indicate that the process of base editing does not significantly perturb steady state mRNA levels, which has implications for determining the safety of base editing in therapeutic settings.

## Discussion

We report here the first systematic study of the cell cycle dependence of both adenine and cytosine BEs. Notably, we observed drastic differences in the mechanism by which Cas9n-derived BEs and dCas9-derived BEs function. Cas9n-derived BEs (which are the most commonly used BE variants) display minimal changes in overall point mutation introduction efficiencies upon synchronization in G1, with small (less than 25% for CBE, and less than 45% for ABE) decreases in efficiency upon synchronization in G2/M. The dCas9-derived BEs both exhibited drastic reductions in their respective point mutation introduction efficiencies upon both G1 (∼65% reductions for ABE and greater than 70% reductions for CBE) and G2/M (∼70% reductions for ABE and ∼80% reductions for CBE) synchronization. These data demonstrate that Cas9n-derived BEs rely on more ubiquitous DNA repair pathways than both dCas9-derived BEs and DSB-reliant technologies. The observation that minimal decreases in both CBE and ABE editing efficiencies occur upon synchronization in G1 is particularly noteworthy, as this is strong mechanistic confirmation that BEs can function well in nondividing cells. The significant decrease in editing efficiencies by dCas9-derived BEs upon both G1 and G2/M synchronization suggests that these tools’ intermediates are highly dependent on S-phase processes to be converted to desired editing outcomes. We suggest that these tools may rely heavily on DNA synthesis across their respective base intermediates to install point mutations. In fact, certain strategies that have been employed to improve HDR-mediated editing by DSB-reliant tools may be effective at improving editing efficiencies by dCas9-derived BEs. These include fusion of Geminin to the editor to enhance its expression levels during S-phase (Gutschner et al., 2016), or fusion of DNA Polymerase D3 (which is involved in genome replication) to the editor (Reint et al., 2021).

Interestingly, we observed drastic increases in C•G to A•T editing efficiencies by CBEΔUGI upon G1 synchronization across multiple cell lines and using different G1 synchronization agents. C•G to non-T•A editing by CBEs has been hypothesized to occur from translesion synthesis (TLS) polymerases processing abasic sites generated from uracil excision by UNG. Our observations may be caused by a combination of cell cycle-dependent changes in UNG and TLS polymerase expression levels, but additional experiments are necessary to further probe this discovery. While the general CBE architecture has recently been repurposed for precision C•G to G•C base editing through DNA repair factor manipulation (Chen et al., 2021), strategies for precision C•G to A•T base editing in mammalian cells do not currently exist. The use of G1 synchronization agents can be used as a starting point to generate more specialized and precise DNA repair manipulation strategies to generate mammalian cell C•G to A•T base editors.

We additionally performed a mechanistic study on base editor-induced indels. An examination of both indel introduction rates and indel sequences introduced by CBE variants show that the introduction of indels by CBEs is dependent on UNG, DNA nicking, and catalytically active deaminase. Specifically, removal of UGI, mutation of H840A in Cas9 (which converts Cas9n to dCas9), or mutation of E58A in rAPOBEC1 (which catalytically inactivates the deaminase) are each independently sufficient to reduce indel introduction rates 10-fold. An analysis of the indel sequences was consistent with these observations as well; CBE-induced indels were found to all be deletion sequences (no insertions were observed, in direct contrast with DSB-reliant genome editing tools). The deletion sequences were either deletions of a single base (a target cytosine) or deletions that spanned the region between the nick and a deaminated cytosine. Taken together, these data suggest CBE-induced indel sequences are likely caused by in situ-generated staggered DSBs, which are putatively formed following processing of UNG-generated abasic sites by endonucleases such as APEX1/2 (which cleave the DNA backbone at abasic sites).

In summary, we have conducted here one of the first mechanistic studies of base editors. We have quantified changes in editing efficiency and precision of both adenine and cytosine base editors with respect to cell cycle synchronization and thus provide key insights into the DNA processing mechanisms of base editor intermediates. Changes in base editing efficiency with respect to cell cycle synchronization suggest nicking BEs rely on more ubiquitous DNA repair pathways than DSB-reliant technologies to introduce their respective point mutations, while non-nicking BEs are highly dependent on S-phase. These results in turn will guide future strategies to enhance base editing efficiency and/or precision and provide more mechanistic details regarding the robustness of nontraditional genome editing agents.

## Materials and Methods

### Constructs and Molecular Cloning

All BE plasmids were constructed with USER cloning (Badran et al., 2016) with pCMV ABEmax_P2A_GFP (Addgene #112101) and pCMV_AncBE4max_P2A_GFP (Addgene #112100) plasmids as template, using Phusion U Hot Start Polymerase (ThermoFisher Scientific). All sgRNA expression plasmids were generated using blunt-end cloning with pFYF1230 (Addgene plasmid #47511) as a template, using Phusion High-Fidelity DNA Polymerase (New England BioLabs). All DNA vector amplification was carried out using NEB 10-β competent cells (New England BioLabs). All plasmids were purified using the ZymoPURE II Plasmid Midiprep Kit (Zymo Research D4200).

### Cell Culture

HEK293T cells (ATCC CRL-3216) were maintained in high glucose DMEM media supplemented with GlutaMAX (ThermoFisher Scientific), 10% (v/v) fetal bovine serum (ThermoFisher Scientific), and 100 µ/ml Penicillin-Streptomycin (ThermoFisher Scientific), at 37°C with 5% CO_2_. K562 cells (ATCC CRL-3344) were maintained in RPMI media (Life Sciences) supplemented as described above.

### Transfections

For all HEK293T cell transfections, 100,000 HEK293T cells in 250 µl of DMEM media without Penicillin-Streptomycin were added per well to 48-well VWR Multiwell Cell Culture Plates on top of lipofectamine/plasmid mixtures. For all K562 cell transfections, 50,000 K562 cells in 250 µl RPMI media without Penicillin-Streptomycin were added per well to 48-well VWR Multiwell Cell Culture Plates on top of lipofectamine/plasmid mixturestransfected at a density 50,000 cells per well in 250 µl RPMI media without Penicillin-Streptomycin. The lipofectamine/plasmid mixtures consisted of 1,000 ng of BE plasmid, 250 ng of sgRNA plasmid, and 1.5 µl of Lipofectamine 2000 (ThermoFisher Scientific) in 25 µl of total volume, made up with Opti-MEM (Gibco #31985-070). Chemical inhibitors were added 6 h after transfection from stock solutions (described below) to result in final concentrations of 5 mM (Thymidine), 800 μM (Mimosine), or 200 ng/ml (Nocodazole).

### Preparation of Synchronizing Agents

Nocodazole (Sigma) was prepared in DMSO to a stock solution concentration of 20 mg/ml. This stock solution was diluted to 50 μg/ml immediately prior to addition to the cells, and 1.1 µl of this diluted stock solution was added to the 275 µl of media in each well, for a final concentration of 200 ng/ml.

Thymidine (Sigma) was prepared in 1X PBS to a stock solution concentration of 50 mM. 30 µl of this stock solution was added to the 275 µl of media in each well, for a final concentration of 5 mM.

Mimosine (Sigma) was prepared in 1X PBS to a stock solution concentration of 10 mM. 24 µl of this stock solution was added to the 275 µl of media in each well, for a final concentration of 800 μM.

Lovastatin (Sigma) was prepared in 95% ethanol to a stock solution concentration of 70 mM with a pH of 7.5. This was diluted to 400 μM, then 30.5 µl of this stock solution was added to the 275 µl of media for a final concentration of 40 μM.

### Flow Cytometry Analysis of Cell Synchronization

3 × 10^5^ HEK293T or K562 cells were plated in a T25 flask in 5 ml of media and synchronizing agents were added to final concentrations as indicated above for 6, 12, or 18 h. Cells were washed with 10 ml PBS, detached with 2 ml of TrypLE (HEK293T cells only), and collected by centrifugation for 10 min at 400 g. Cells were resuspended at 1 × 10^6^ cells/ml in 1 ml cold PBS, added to 9 ml cold 70% ethanol, and stored for at least 4 h at −20°C. After ethanol fixation, cells were centrifuged at 400 g, washed with 10 ml cold PBS, then stained with 400 µl PI solution [0.1% Triton X-100 (Sigma), 0.2 mg/ml RNAse (Sigma), 0.02 mg/ml PI (Sigma) in PBS]. Cells were incubated at 37°C for 15 min prior to analysis. Cells were gated to exclude doublets and non-viable cells. Fluorescent signal from PI staining was analyzed *via* histogram on either a BLDSRFortessa or BioRad S3e cell sorter.

### Flow Cytometry Analysis of Green Fluorescent Protein Fluorescence

For all GFP fluorescence measurements, 1 × 10^6^ cells were resuspended in FACS buffer [1% FBS, 50 µM EDTA pH 8.0, 2 μg/ml PI (Sigma)] and filtered through a cell-strainer. Non-viable cells and doublets were eliminated *via* gating parameters. Flow cytometry was performed on a BioFortessa or S3e cell sorter (Bio-Rad).

### Transfection Efficiency Quantification

Transfection efficiency was determined *via* flow cytometry analysis of cells 24 h post-transfection. Chemical inhibitors were added to HEK293T cells at −17, 0, and 6 h relative to transfection of BE and gRNA plasmids (as described above). 24 h post-transfection, cells were washed with PBS, detached from the plate with 50 µl Accutase (Innovative Cell Technologies), resuspended in 250 µl FACS buffer, and analyzed by flow cytometry as described above. The percent of cells with GFP fluorescence was analyzed *via* Flowjo.

### High-Throughput DNA Sequencing of Genomic DNA

Transfected cells were rinsed with 150 µl PBS (ThermoFisher Scientific) per well at the indicated time points after transfection. Cells were lysed on the plate by addition of 100 µl of lysis buffer (10 mM Tris, pH 7.5, 0.1% SDS, and 25 μg/ml Proteinase K). Lysed cells were then heated at 37°C for 1 h, followed by 80°C for 20 min. Genomic loci of interest were PCR amplified with Phusion High-Fidelity DNA Polymerase (New England BioLabs) according to the manufacturer’s protocol, with primers bearing homology to the target site and relevant Illumina forward and reverse adapters (Supplementary Table S2), 1 µl of genomic DNA mixture as a template, and 26 or fewer rounds of amplification. Unique forward and reverse combinations of Illumina adapter sequences were then appended with an additional round of PCR amplification with Phusion High-Fidelity DNA Polymerase (New England BioLabs) according to the manufacturer’s protocol, using 1 µl of round 1 PCR mixture as a template and 15 rounds of amplification. The products were gel purified from 2% agarose gel with QIAquick Gel Extraction Kit (Qiagen) and quantified using NEBNext Ultra II DNA Library Prep Kit (NEB) on a CFX96 system (BioRad). Samples were then sequenced on an Illumina MiniSeq according to the manufacturer’s protocol.

### High-Throughput DNA Sequencing and Indel Analysis of Targeted Amplicon Sequencing Reads

Analysis of Illumina HTS sequencing readout was conducted with CRISPRessov2 (Komor et al., 2016; Clement et al., 2019). Specifically, for these analyses, fastq files were analyzed *via* scripts run on Docker, where the reads were analyzed against the entire amplicons, with outputs for the guide RNA and base editor (--guide_seq and –base_editor_output). Product distribution for CBE variants was determined by taking the fraction of individual A•T, G•C, and T•A reads and dividing by the sum. CRISPResso was also used to validate editing percentages and analyze indel frequency, where the total number of indel reads was obtained from the indel histogram output and expressed as the fraction of reads with indel over total reads. For analysis of indel sequences, specific reads constituting >4% of the total indels from the CRISPResso were compiled.

### mRNA Sequencing Experiments

For mRNA sequencing experiments, cells were transfected in 48-well plates at 150,000 cells/well with ABE 7.10 F148A, ABE 7.10 F148A E59A, BE4-W90Y-R126E, BE4-W90Y-R126E-E63A, BE4-W90Y-R126E-ΔUGI or BE4-W90Y-R126E-E63A-ΔUGI, together with plasmids expressing HEK2 or non-targeting gRNA. Samples were lysed at 48 h post-transfection. Both gDNA and total RNA were extracted in separate replicate samples. Genomic DNA was extracted as previously described.

### RNA Isolation and Purification

Total RNA was isolated *via* Zymo RNA extractions kit (Zymo, R1054), as per manufacturer’s protocol. Cells were lysed with 300 µl RNA lysis buffer before addition of 300 µl 100% ethanol. The mixture was centrifuged in the Zymo-Spin IC Column at 16,000 rcf for 30 s. DNase treatment followed this by washing with 400 µl RNA wash buffer and treating with 5 µl DNase 1 (1 µ/µl) and 35 µl DNA Digestion Buffer for 15 min at room temperature. 400 µl RNA Prep Buffer was added to the column, centrifuged, and washed 2x with RNA wash buffer prior to collection with 50 µl DNase/RNase-Free water. RNA was stored at −20°C before library preparation.

### RNA-Sequencing

For HEK293T cell lines, total RNA was assessed for quality using an Agilent Tapestation 4200, and samples with an RNA Integrity Number (RIN) greater than 8.0 were used to generate RNA sequencing libraries using the TruSeq Stranded mRNA Sample Prep Kit with TruSeq Unique Dual Indexes (Illumina, San Diego, CA, United States). Samples were processed following manufacturer’s instructions, starting with 500 ng of RNA and modifying RNA shear time to 5 min. Quality of resulting libraries was assessed by Agilent Tapestation 4200, and libraries were multiplexed and sequenced with 100 basepair (bp) paired end reads (PE100) to a depth of approximately 25 million reads per sample on an Illumina NovaSeq 6000. Samples were demuxltiplexed using bcl2fastq v2.20 Conversion Software (Illumina, San Diego, CA, United States).

### RNA-Sequencing Analysis

All fastq files were trimmed using Trimmomatic (https://github.com/usadellab/Trimmomatic) using the Illumina PE adapters. The trimmed reads were assessed with FastQC (https://github.com/s-andrews/FastQC) and then passed through the following analytical pipeline: transcript pseudoalignment and quantification was performed with Salmon (https://github.com/COMBINE-lab/salmon) using an index generated from the GENCODE version 32 transcriptome using standard arguments, trimmed reads were aligned to a *Homo sapiens* genome assembly GRCh38 (hg38) using STAR (https://github.com/alexdobin/STAR) with default arguments using a previously described 2-pass approach. Salmon output was imported into a DESeq object using tximport and differential expression analysis was performed with DESeq2 using standard arguments. Differentially expressed genes were called with FDR-corrected p (p-adj) ≤0.01 and fold change >2 cutoffs, and results were visualized in R.

### Gene Set Enrichment Analysis

Genes determined to be differentially expressed in DESeq2 output were ranked [score metric = sin (log2FoldChange) * −log (*p*-value)] and processed using the R package *fgsea* in conjunction with gene set files downloaded from MSigDB using the package *msgdbr*. Additional code was written for select visualizations of DNA damage repair genes.

## Data Availability

The datasets presented in this study can be found in online repositories. The names of the repository/repositories and accession number(s) can be found in the article/Supplementary Material.
