# Supplementary material for: Examination of the Cell Cycle Dependence of Cytosine and Adenine Base Editors
Source: Front Genome Ed. 2022 Jul 14;4:923718. doi: 10.3389/fgeed.2022.923718 (PMC9333457; doi:10.3389/fgeed.2022.923718)
Supplement: Supplementary file 1 [file DataSheet1.pdf]

Supplementary Information for:

Examination of the Cell Cycle Dependence of Cytosine and Adenine Base Editors

Cameron A. Burnett[1], Ashley T. Wong[1], Carlos A. Vasquez[1], Colleen A. McHugh[1], Gene W. Yeo[2], and Alexis C. Komor[1*]

Data availability statement	Repository and accession numbers of datasets
Supplementary Figure 1	Time course of base editing by ABE, CBE, and CBEΔUGI
Supplementary Figure 2	Cell synchronization quantification
Supplementary Figure 3	Synchronization effects on HEK293T cell viability and BE expression levels
Supplementary Figure 4	Synchronization of HEK293T cells in following transfection in 48-well format
Supplementary Figure 5	Cell cycle synchronization effects on base editing efficiencies and precision of Cas9n-derived BEs in K562 cells
Supplementary Figure 6	Cell cycle synchronization effects on base editing efficiencies by dCas9-derived BEs in K562 cells
Supplementary Figure 7	Product distribution of CBEs in HEK293T cells
Supplementary Figure 8	Product distribution of CBEΔUGI upon G1 synchronization at additional genomic loci
Supplementary Figure 9	Effects on CBEΔUGI product distribution by G1 synchronization with mimosine in HEK293T cells
Supplementary Figure 10	Cell cycle synchronization effects on base editing precision of dCas9-derived CBEs in HEK293T cells
Supplementary Figure 11	Effects of cell cycle synchronization and catalytic inactivation of the deaminase on indel introduction efficiencies in HEK293T cells
Supplementary Figure 12	Cell viability of HEK293T cells treated with BEs and HEK2 gRNAs
Supplementary Figure 13	Hits from differential expression analysis of HEK293T cells undergoing base editing at the *HEK2* (H2) genomic locus by RNA off-target optimized constructs
Supplementary Sequences	Protein sequences of all constructs used in this work
Supplementary Table 1	List of DNA repair genes categorized by DNA repair pathways (in separate excel file)
Supplementary Table 2	Protospacer and PAM sequences for all sites used for assessing editing efficiencies
Supplementary Table 3	First round genomic DNA PCR primer sequences

Data availability statement

High-throughput sequencing data have been deposited in the NCBI Sequencing Read Archive database under Accession Number PRJNA854675.

Supplementary Figures

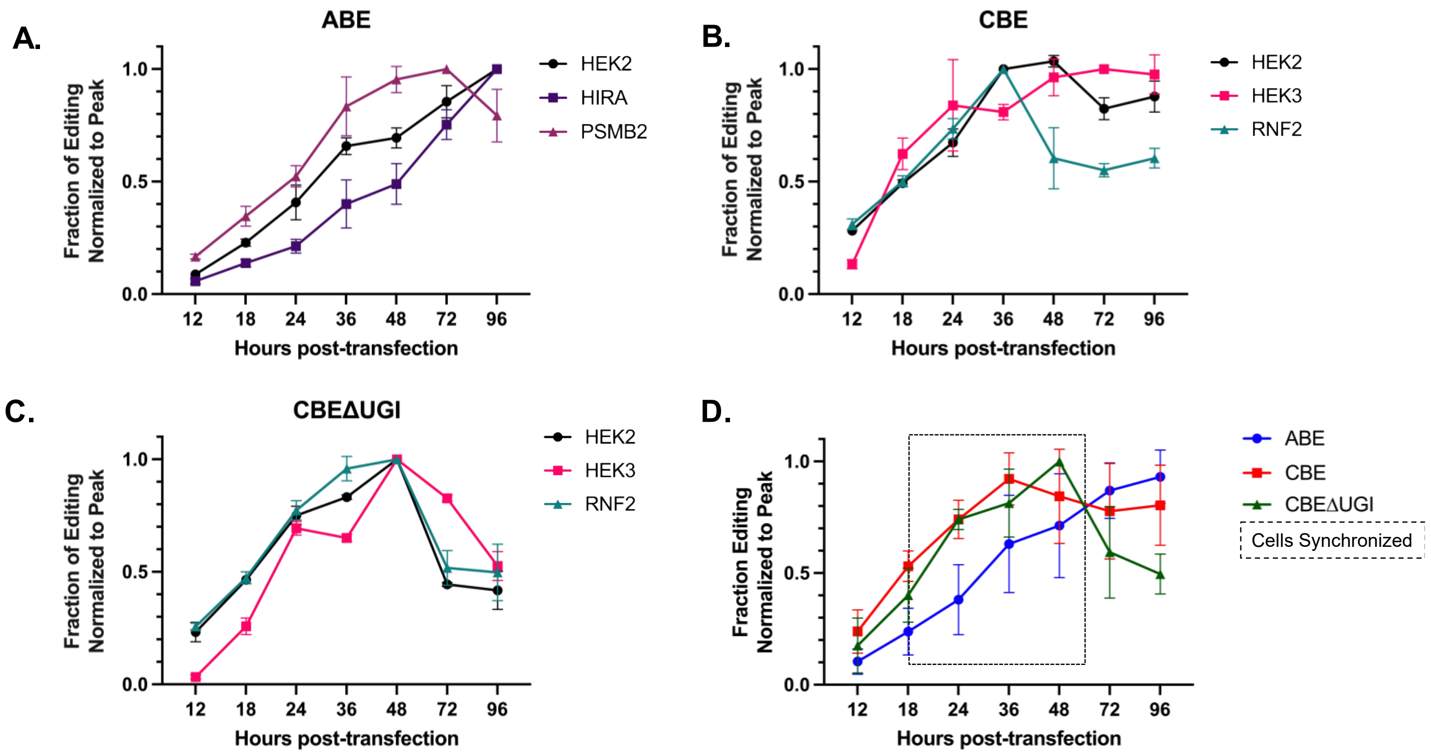

Supplementary Figure 1. Time course of base editing by ABE, CBE, and CBEΔUGI. (A, B, C) HEK293T cells were transfected with ABE or CBE plus gRNA (protospacer sequences indicated in Figure 1), and lysed at the indicated time points. The genomic DNA was extracted, and target loci were amplified via PCR and subjected to high-throughput sequencing (HTS). Genome editing efficiencies (percent of total HTS reads with the target A•T base converted to G•C for ABE, percent of total HTS reads with the target C•G base converted to T•A for CBE, or percent of total HTS reads with the target C•G base converted to T•A, G•C, or A•T for CBEΔUGI) were quantified with CRISPResso2. Efficiencies were then normalized to the maximum efficiency observed for a given protospacer over the course of the experiment. Shown are results from individual genomic loci for ABE (A), CBE (B), and CBEΔUGI (C). We additionally averaged these normalized efficiencies across all three sites for ABE, CBE, and CBEΔUGI (D). Values and error bars reflect the means and SD of three independent biological replicates performed on different days.

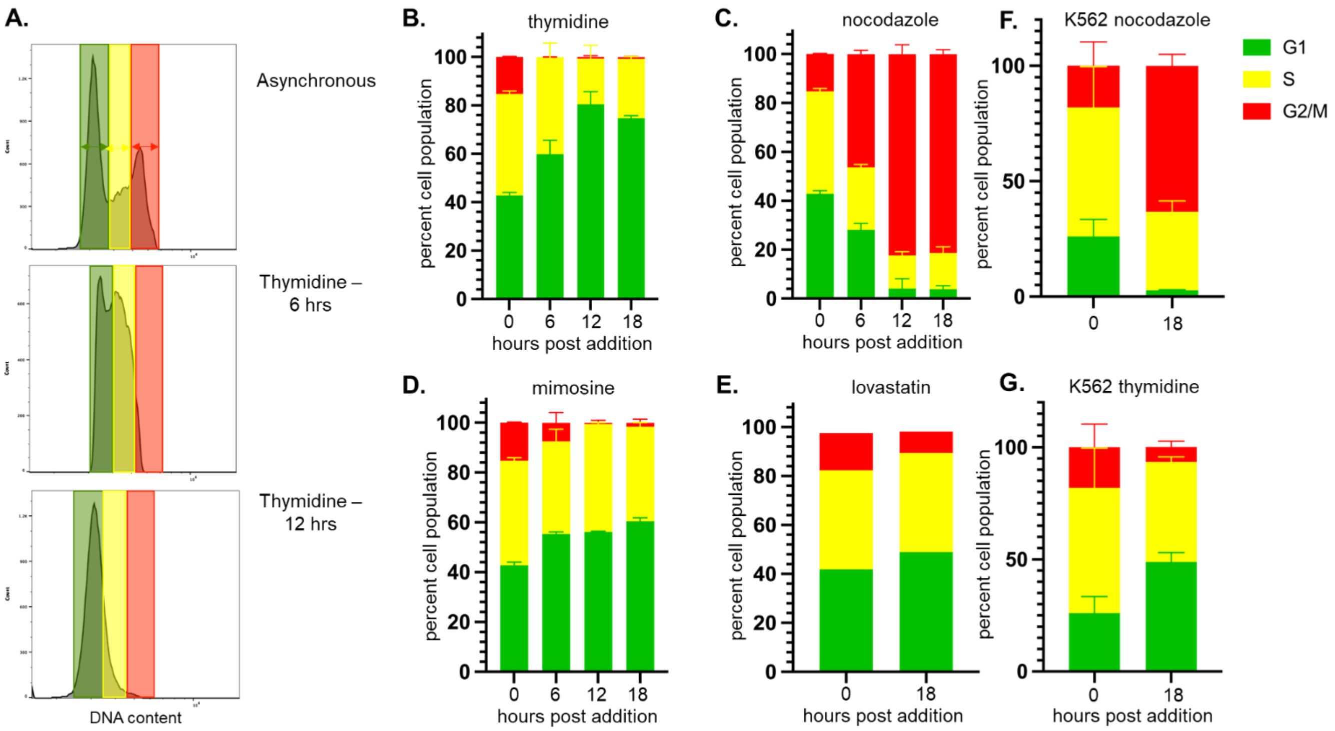

Supplementary Figure 2. Cell synchronization quantification. HEK293T cells (A-E) or K562 cells (F-G) were treated with chemical inhibitors for 6, 12, and 18 hours and stained with propidium iodide (PI) after ethanol fixation. The percent of the total cell population in G1, S, or G2/M was determined based off of PI fluorescence level, which quantifies DNA content. Representative PI stain plots are shown in (A), and quantification is shown for Thymidine (B and G), Nocodazole (C and F), Mimosine (D), and Lovastatin (E). Values and error bars reflect the means and SD of three independent biological replicates performed on different days, except for lovastatin which represents a single biological replicate.

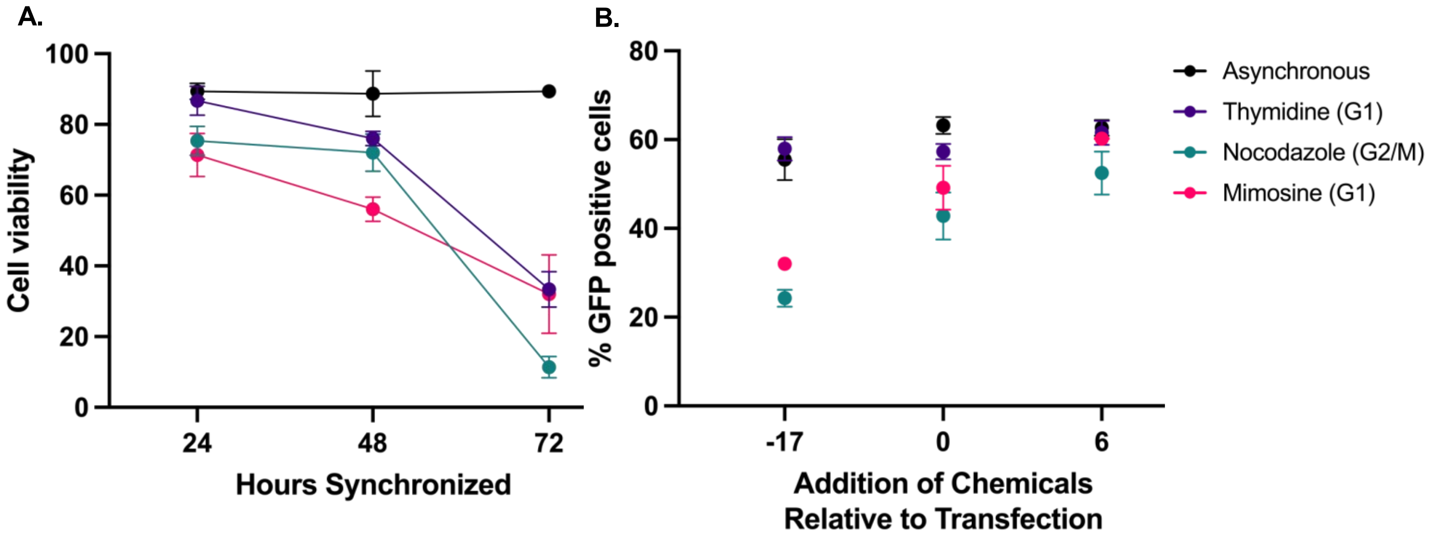

Supplementary Figure 3. Synchronization effects on HEK293T cell viability and BE expression levels. (A) HEK293T cell viability, as assessed via Trypan blue stain, relative to length of time that cells are held synchronized. (B) Percent of HEK293T cells with GFP fluorescence relative to the time that synchronization agents were added (with respect to transfection of BE and gRNA). Cell were transfected with ABE-P2A-GFP plus gRNA and analyzed 24 hours post-transfection for GFP expression via flow cytometry.

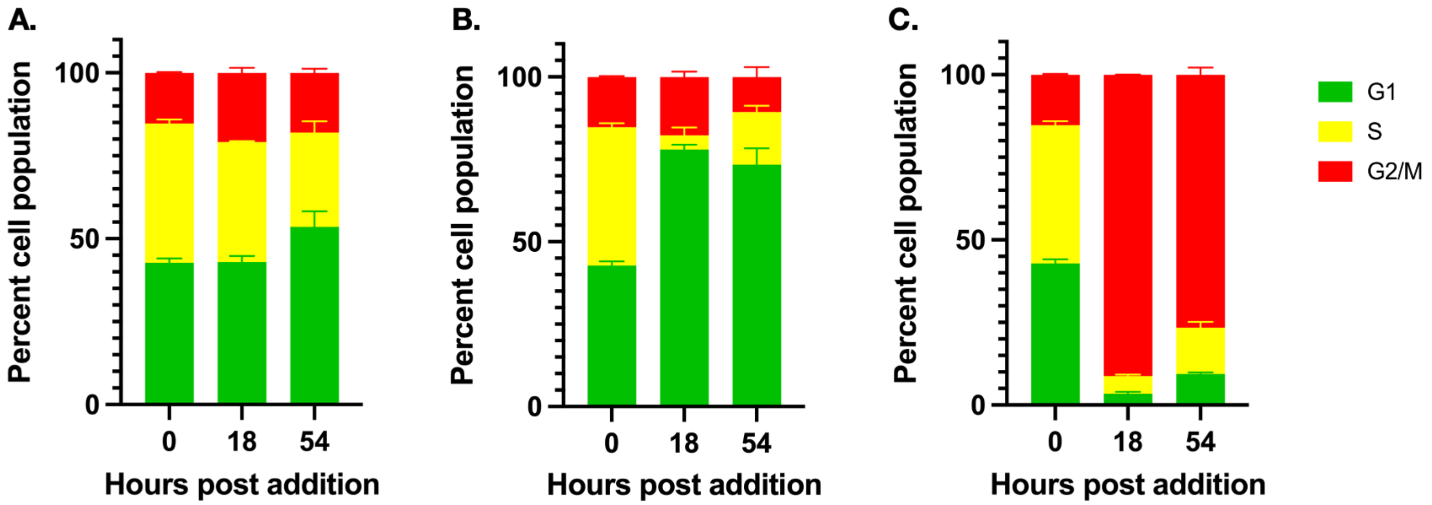

Supplementary Figure 4. Synchronization of HEK293T cells in following transfection in 48-well format. (A) Asynchronous HEK293T cells were transfected with ABE-P2A-GFP plus gRNA and analyzed for DNA content via ethanol fixation and PI staining at 0, 18 and 54 hours. (B) Transfected HEK293T cells treated with thymidine. (C) Transfected HEK293T cells treated with nocodazole.

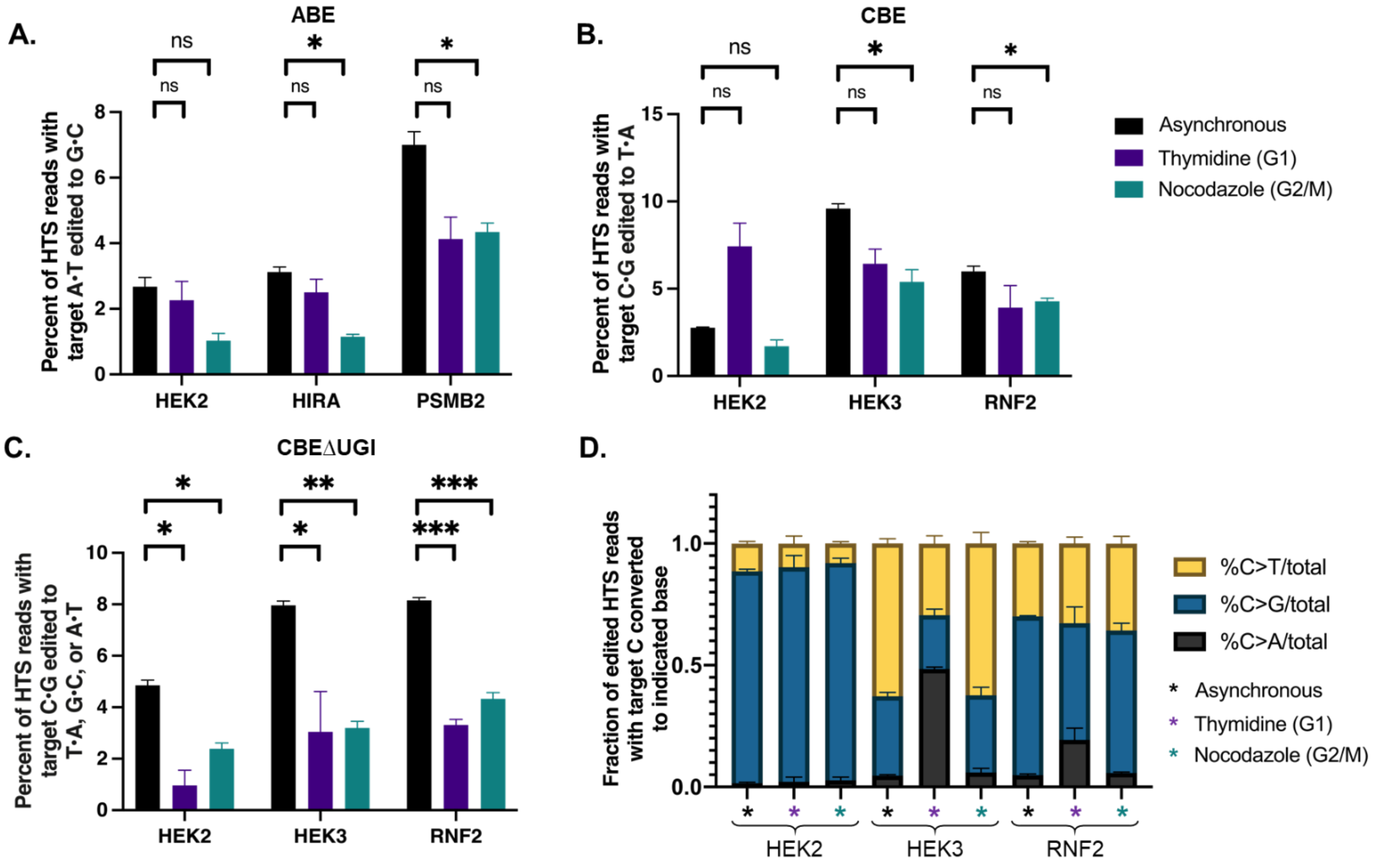

Supplementary Figure 5. Cell cycle synchronization effects on base editing efficiencies and precision of Cas9n-derived BEs in K562 cells. Cells were transfected with ABE (A), CBE (B), or CBEΔUGI (C and D) plus gRNA (protospacer sequences indicated in Figure 1), synchronization agents were added 6 hours post-transfection (thymidine for G1 synchronization or nocodazole for G2/M synchronization), and cells were lysed at 54 hours. The genomic DNA was extracted, and target loci were amplified via PCR and subjected to HTS. Genome editing efficiencies (percent of total HTS reads with the target A•T base converted to G•C for ABE, percent of total HTS reads with the target C•G base converted to T•A for CBE, or percent of total HTS reads with the target C•G base converted to T•A, G•C, or A•T for CBEΔUGI) were quantified with CRISPResso2. Base editing efficiencies by ABE (A), CBE (B), and CBEΔUGI (C) upon synchronization are plotted. (D) The product distribution, defined as the relative portion of edited sequencing reads (reads in which the target C•G is mutated to T•A, A•T, or G•C) that have been edited to each of the indicated outcomes, is plotted for CBEΔUGI. Values and error bars reflect the means and SD of three independent biological replicates performed on different days. Asterisks reflect p value calculations of unpaired *t* test, one tailed (ns indicates not significant, *$p<0.05$, **$p<0.01$, ***$p<0.001$).

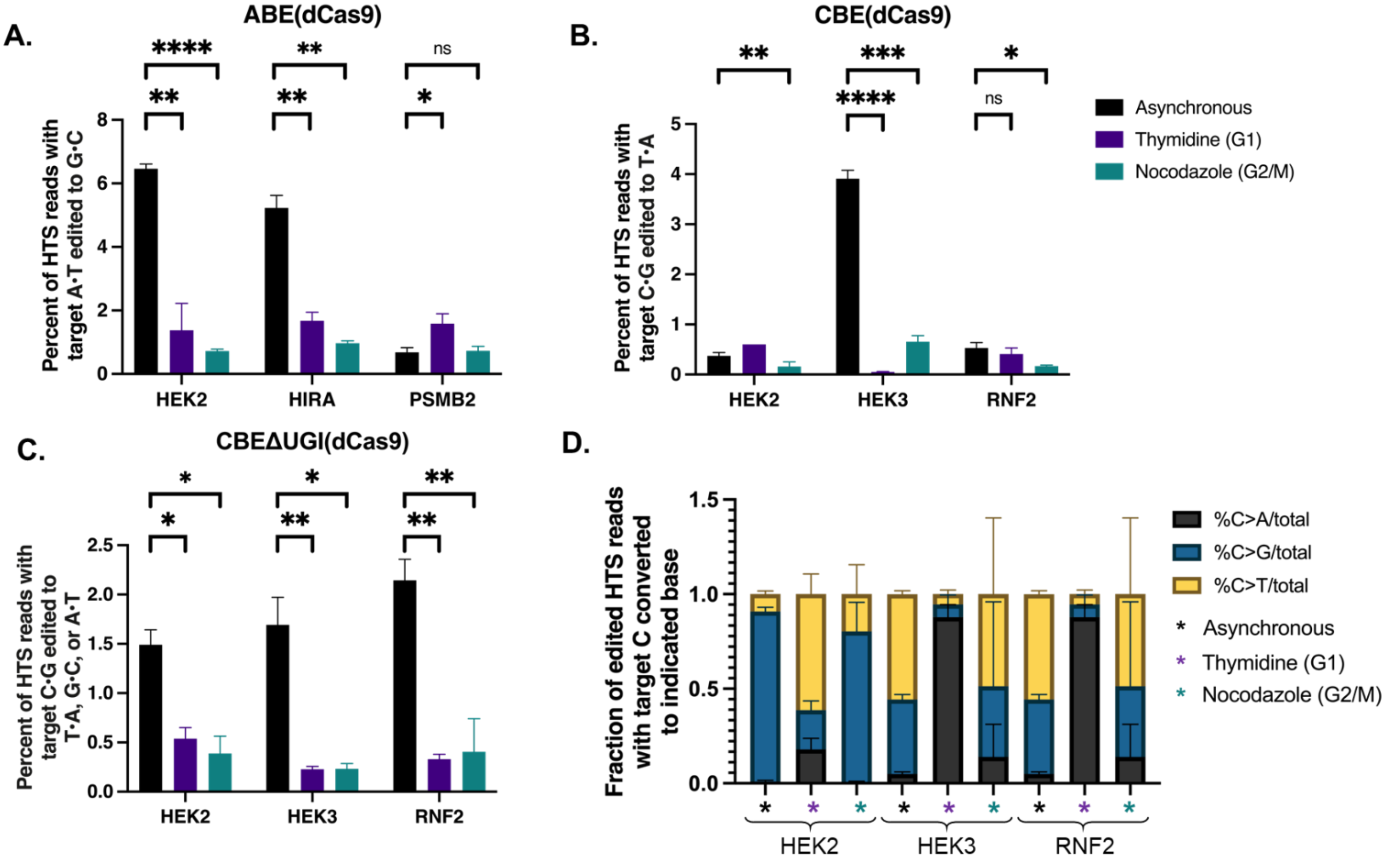

Supplementary Figure 6. Cell cycle synchronization effects on base editing efficiencies by dCas9-derived BEs in K562 cells. Cells were transfected with ABE(dCas9), CBE(dCas9), or CBEΔUGI(dCas9) plus gRNA (protospacer sequences indicated in Figure 1), synchronization agents were added 6 hours post-transfection (thymidine for G1 synchronization or nocodazole for G2/M synchronization), and cells were lysed at 54 hours. The genomic DNA was extracted and target loci were amplified via PCR and subjected to HTS. Genome editing efficiencies (percent of total HTS reads with the target A•T base converted to G•C for ABE(dCas9), percent of total HTS reads with the target C•G base converted to T•A for CBE(dCas9), or percent of total HTS reads with the target C•G base converted to T•A, G•C, or A•T for CBEΔUGI) were quantified with CRISPResso2. Base editing efficiencies by ABE(dCas9) (A), CBE(dCas9) (B), and CBEΔUGI(dCas9) (C) upon synchronization are plotted. (D) The product distribution, defined as the relative portion of edited sequencing reads (reads in which the target C•G is mutated to T•A, A•T, or G•C) that have been edited to each of the indicated outcomes, is plotted for CBEΔUGI(dCas9). Values and error bars reflect the means and SD of three independent biological replicates

performed on different days. Asterisks reflect p value calculations of unpaired *t* test, one tailed (ns indicates not significant, **p*<0.05, ***p*<0.01, ****p*<0.001, ****p<0.0001).

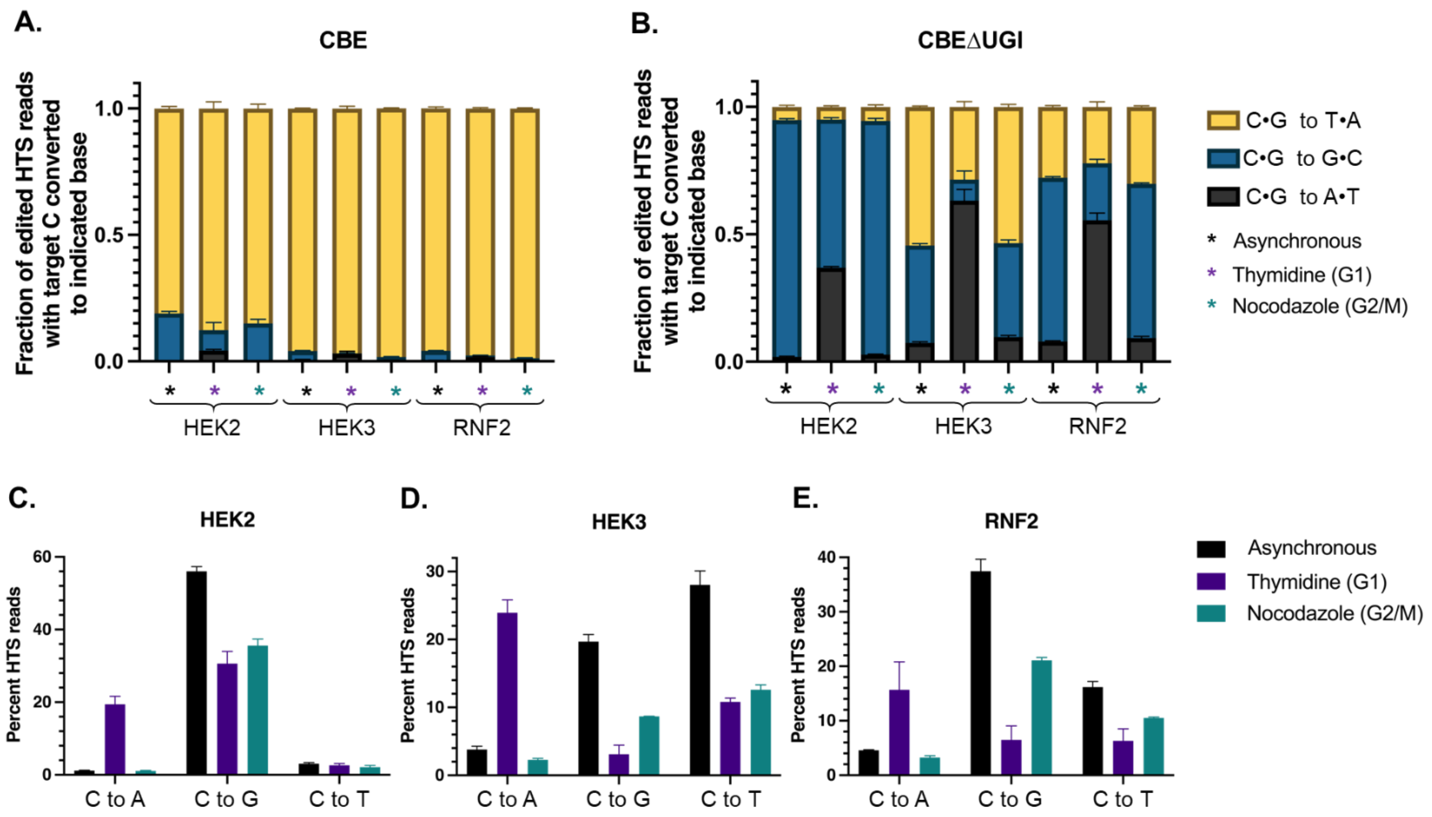

Supplementary Figure 7. Product distribution of CBEs in HEK293T cells. HEK293T cells were transfected with CBE (A) or CBEΔUGI (B, C, D, E) plus gRNA (protospacer sequences indicated in Figure 1), synchronization agents were added 6 hours post-transfection (thymidine for G1 synchronization or nocodazole for G2/M synchronization), and cells were lysed at 54 hours. The genomic DNA was extracted and target loci were amplified via PCR and subjected to HTS. Genome editing efficiencies (percent of total sequencing reads with target C•G mutated to T•A, A•T, or G•C) were quantified with CRISPResso2. (A, B) The product distribution, defined as the relative portion of edited sequencing reads (reads in which the target C•G is mutated to T•A, A•T, or G•C) that have been edited to each of the indicated outcomes, is plotted for CBE (A) and CBEΔUGI (B). The data in (B) is also presented in **Figure 3B**. (C, D, E) Absolute editing efficiencies (percent of total sequencing reads with target C•G mutated to T•A, A•T, or G•C) are plotted for CBEΔUGI at each target site. Values and error bars reflect the means and SD of three independent biological replicates performed on different days.

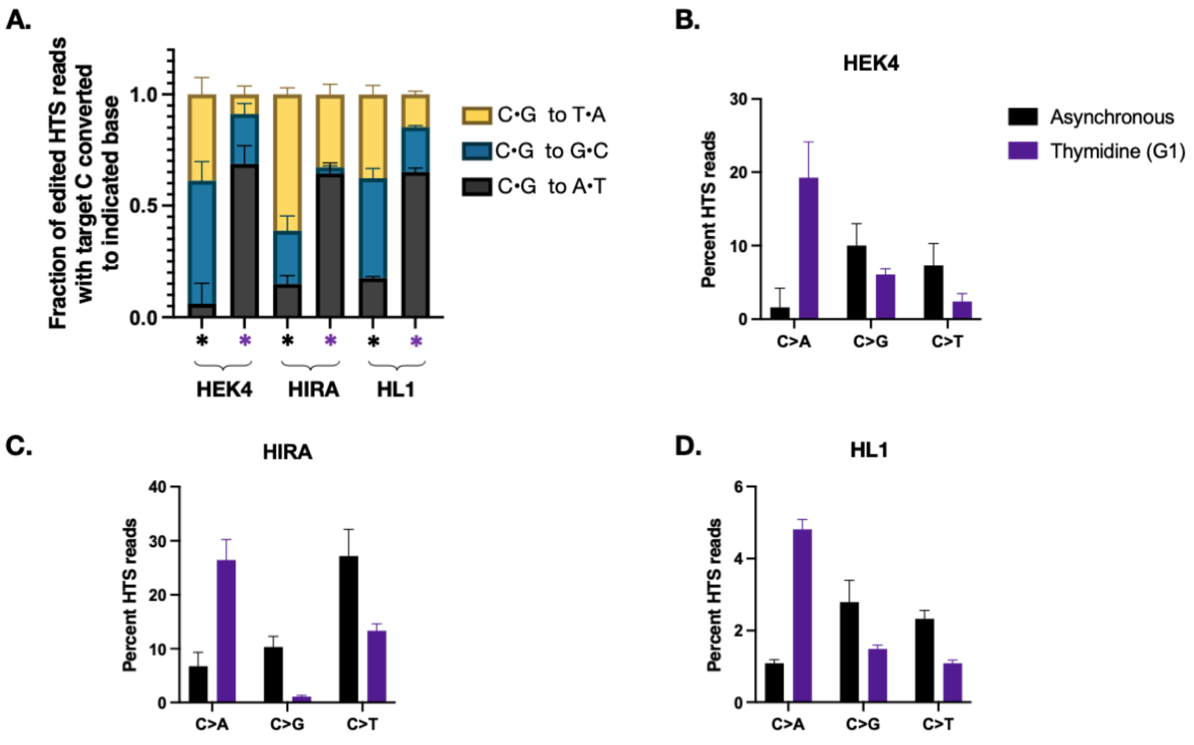

Supplementary Figure 8. Product distribution of CBEΔUGI upon G1 synchronization at additional genomic loci. HEK293T cells were transfected CBEΔUGI plus gRNA (protospacer sequences indicated in Supplementary Table 2), synchronization agent (thymidine) was added 6 hours post-transfection, and cells were lysed at 54 hours. The genomic DNA was extracted and target loci were amplified via PCR and subjected to HTS. Genome editing efficiencies (percent of total sequencing reads with target C•G mutated to T•A, A•T, or G•C) were quantified with CRISPResso2. (A) The product distribution, defined as the relative portion of edited sequencing reads (reads in which the target C•G is mutated to T•A, A•T, or G•C) that have been edited to each of the indicated outcomes, is plotted for CBEΔUGI. (B, C, D) Absolute editing efficiencies (percent of total sequencing reads with target C•G mutated to T•A, A•T, or G•C) are plotted for CBEΔUGI at each target site.

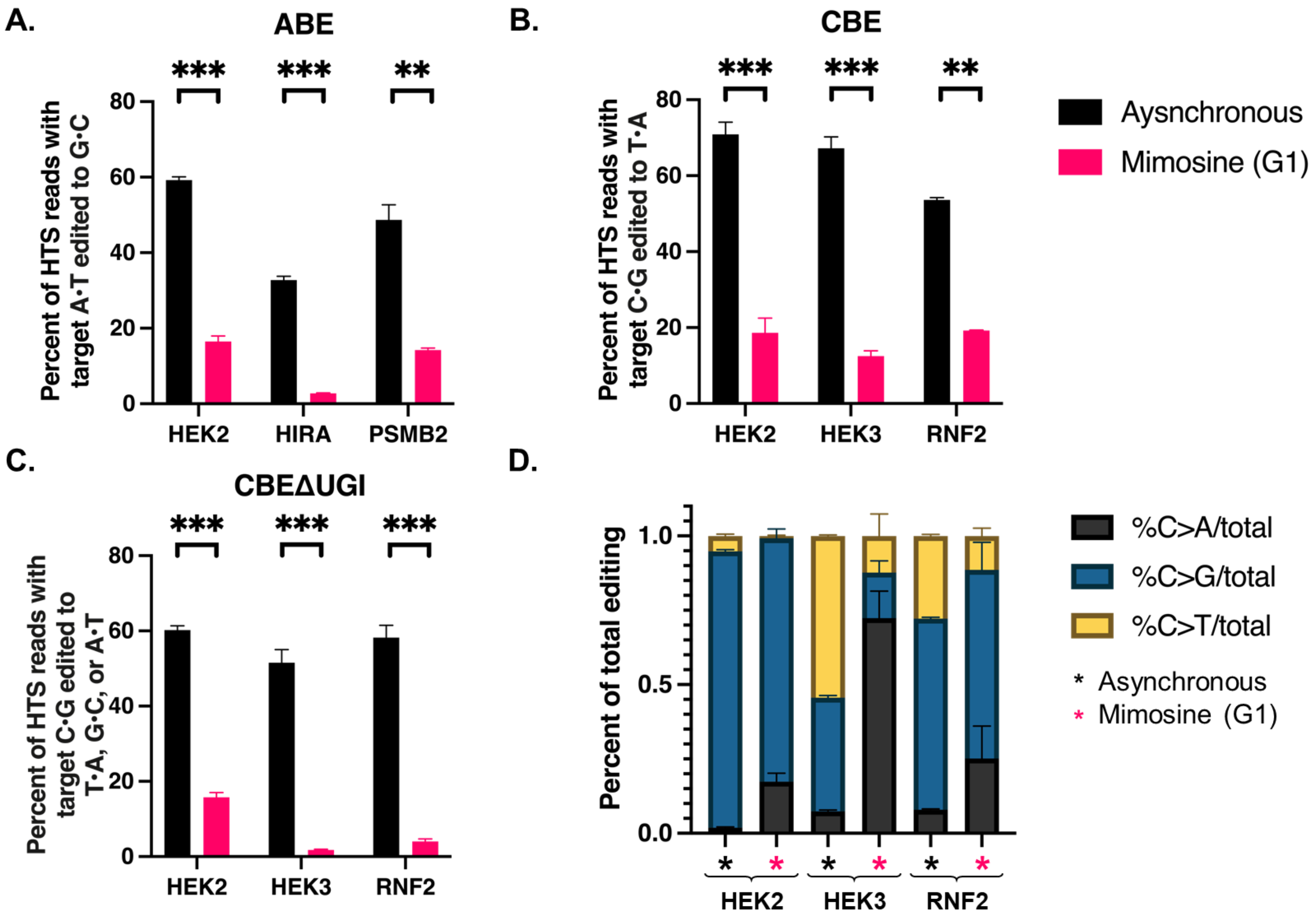

Supplementary Figure 9. Effects on CBEΔUGI product distribution by G1 synchronization with mimosine in HEK293T cells. HEK293T cells were transfected with ABE, CBE, or CBEΔUGI plus gRNA (protospacer sequences indicated in Figure 1), mimosine was added 6 hours post-transfection (for G1/S synchronization), and cells were lysed at 54 hours. The genomic DNA was extracted, and target loci were amplified via PCR and subjected to HTS. Genome editing efficiencies (percent of total HTS reads with the target A•T base converted to G•C for ABE, percent of total HTS reads with the target C•G base converted to T•A for CBE, or percent of total HTS reads with the target C•G base converted to T•A, G•C, or A•T for CBEΔUGI) were quantified with CRISPResso2. Base editing efficiencies by ABE (A), CBE (B), and CBEΔUGI (C) upon synchronization are plotted. (D) The product distribution, defined as the relative portion of edited sequencing reads (reads in which the target C•G is mutated to T•A, A•T, or G•C) that have been edited to each of the indicated outcomes, is plotted for CBEΔUGI. Values and error bars reflect the means and SD of three independent biological replicates

performed on different days. Asterisks reflect p value calculations of unpaired *t* test, one tailed (ns indicates not significant, *$p<0.05$, **$p<0.01$, ***$p<0.001$).

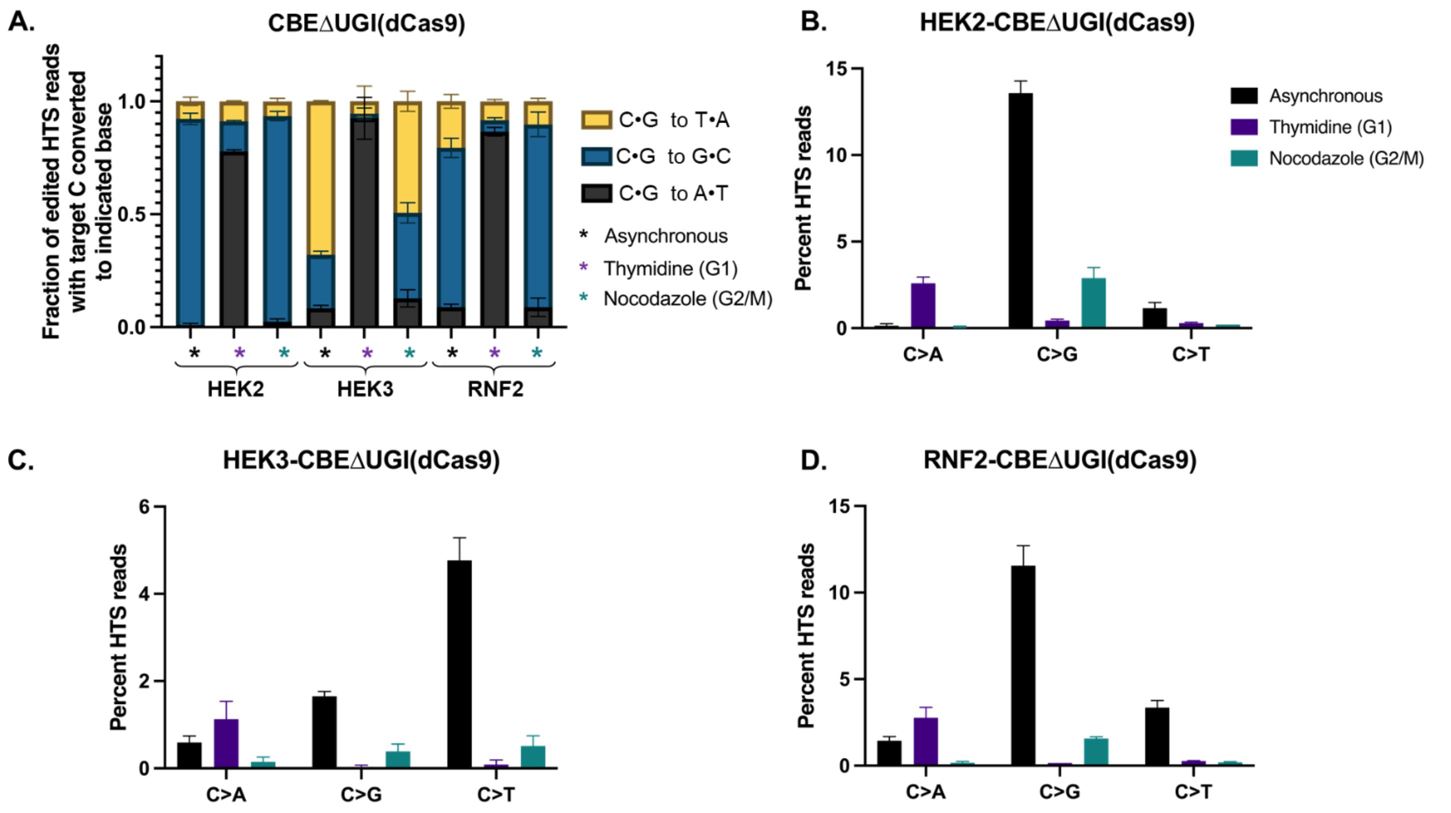

Supplementary Figure 10. Cell cycle synchronization effects on base editing precision of dCas9-derived CBEs in HEK293T cells. HEK293T cells were transfected with CBEΔUGI(dCas9) plus gRNA (protospacer sequences indicated in Figure 1), synchronization agents were added 6 hours post-transfection (thymidine for G1 synchronization or nocodazole for G2/M synchronization), and cells were lysed at 54 hours. The genomic DNA was extracted, and target loci were amplified via PCR and subjected to HTS. Genome editing efficiencies (percent of total HTS reads with the target A•T base converted to G•C for ABE, percent of total HTS reads with the target C•G base converted to T•A for CBE, or percent of total HTS reads with the target C•G base converted to T•A, G•C, or A•T for CBEΔUGI) were quantified with CRISPResso2. (A) The product distribution, defined as the relative portion of edited sequencing reads (reads in which the target C•G is mutated to T•A, A•T, or G•C) that have been edited to each of the indicated outcomes, is plotted for CBEΔUGI. The data in (A) is also presented in **Figure 3D**. Absolute editing efficiencies (percent of total sequencing reads with target C•G mutated to T•A, A•T, or G•C) are plotted for CBEΔUGI at each target site (B,C,D). Values and error bars reflect the means and SD of three independent biological replicates performed on different days.

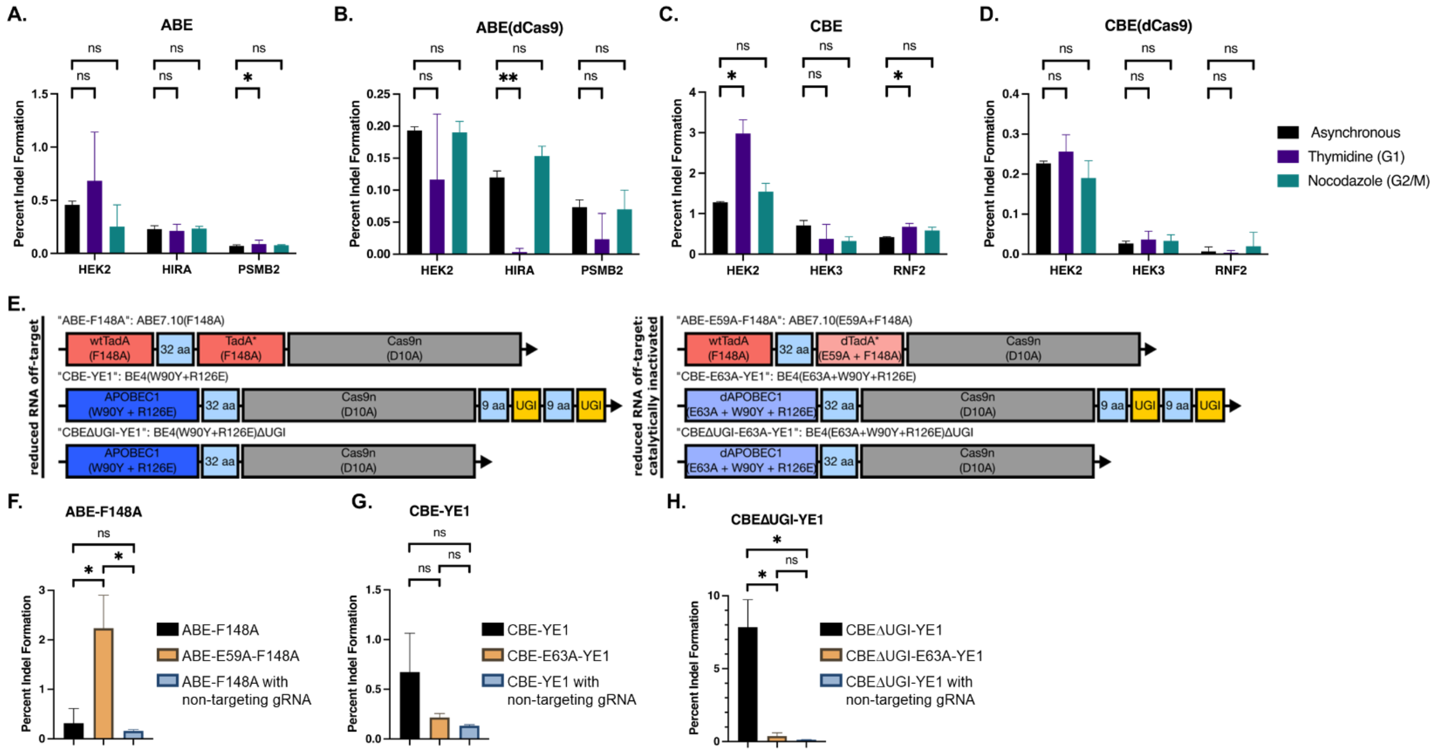

Supplementary Figure 11. Effects of cell cycle synchronization and catalytic inactivation of the deaminase on indel introduction efficiencies in HEK293T cells. In (A) through (D), HEK293T cells were transfected with ABE (A), ABE(dCas9) (B), CBE (C), or CBE(dCas9) (D) plus gRNA (protospacer sequences indicated in Figure 1), synchronization agents were added 6 hours post-transfection (thymidine for G1 synchronization or nocodazole for G2/M synchronization), and cells were lysed at 54 hours. The genomic DNA was extracted and target loci were amplified via PCR and subjected to HTS. Total indel introduction efficiencies were calculated as the percent of reads with insertions or deletions (determined via CRISPResso analysis) divided by the total number of HTS reads sequenced. The data in (C) is also presented in **Figure 4A** for comparison purposes. (E) Construct maps and names of reduced-RNA editing base editor variants. In (F) through (H), HEK293T cells were transfected with the reduced-RNA editing variants ABE-F148A (F), CBE-YE1 (G), CBEΔUGI-YE1 (H), or their catalytically inactivated deaminase counterparts ABE-E59A-F148A (F), CBE-E63A-YE1 (G), CBEΔUGI-E63A-YE1 (H) and either a HEK2-targeting gRNA or a non-targeting gRNA. Cells were lysed 48 hours after transfection, the genomic DNA was extracted, and target loci were amplified via PCR and subjected to HTS. Total indel introduction efficiencies were calculated as the percent of reads with insertions or deletions (determined via CRISPResso analysis) divided by the total number of HTS reads sequenced. Values and error bars reflect the

means and SD of three independent biological replicates performed on different days. Asterisks reflect p value calculations of unpaired *t* test, one tailed (ns indicates not significant, *$p<0.05$, **$p<0.01$).

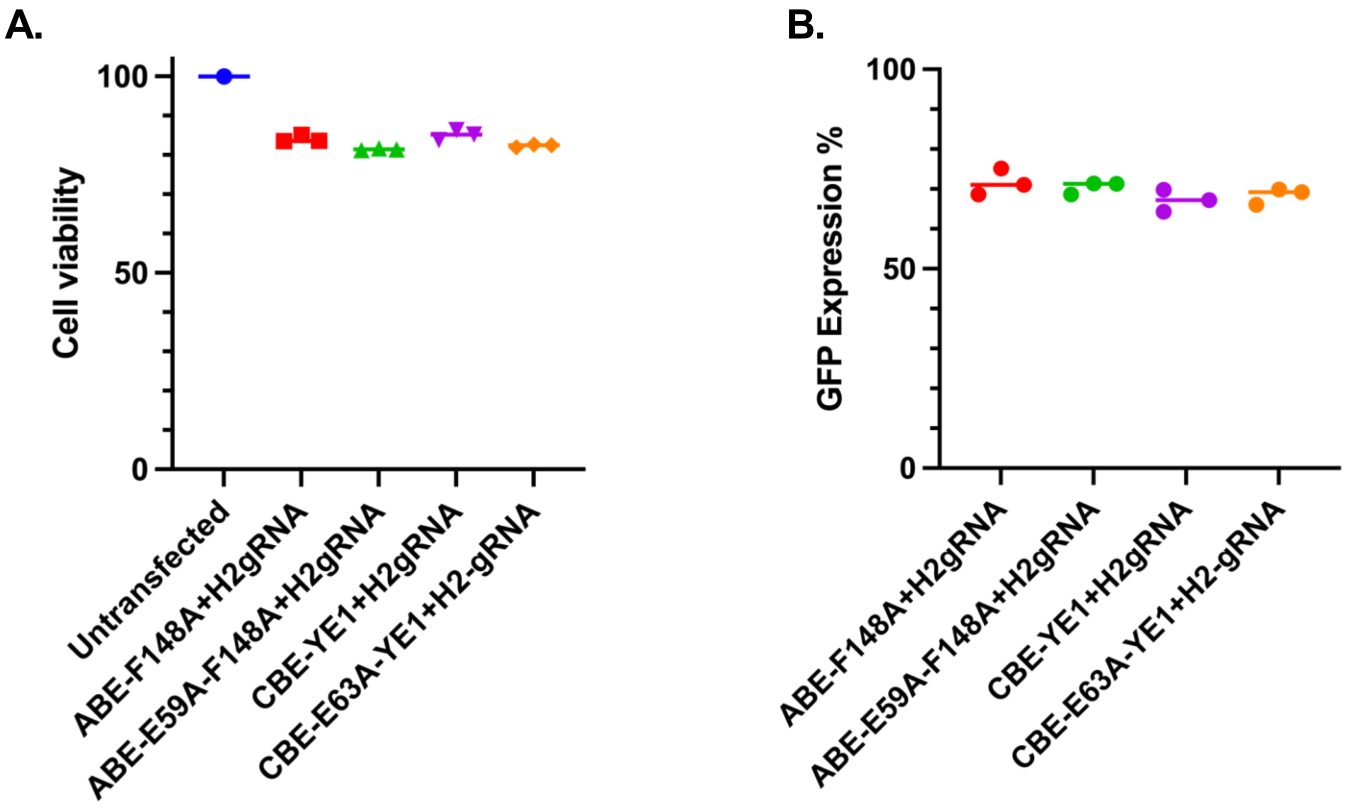

Supplementary Figure 12. Cell viability of HEK293T cells treated with BEs and HEK2 gRNAs. HEK293T cells were transfected with the reduced-RNA editing variants ABE-F148A or CBE-YE1, or their catalytically inactivated deaminase counterparts ABE-E59A-F148A or CBE-E63A-YE1, CBEΔUGI-E63A-YE1 and a HEK2-targeting gRNA (H2gRNA). (A) At 48 hours post-transfection, cells were stained with 2 µg/mL propidium iodine to assess cell viability. Viability was determined to be >75% for cells lysed and extracted for RNAseq in Figure 5. (B) Also at 48 hours post-transfection, GFP fluorescence was measured by flow cytometry to assess BE expression levels.

comparison	up-/down-regulated	gene	function
ABE-F148A H2-gRNA vs. ABE-F148A nt-gRNA	up	AC011511.4	Long non-coding RNA, potentially subject of nonsense-mediated decay
ABE-F148A H2-gRNA vs. ABE-E59A-F148A H2-gRNA	up	HSPA6	Heat shock 70 kDa protein 6: molecular chaperone involved in protein quality control
CBE-YE1 H2-gRNA vs. CBE-YE1 nt-gRNA	up	RPS3AP26	Ribosomal Protein S3a Pseudogene 26
	down	AC016026.1	Long intergenic non-coding RNA; function unknown
	down	CXCR2	C-X-C chemokine receptor type 2: interleukin-8 receptor, causes activation of neutrophils
	down	ANXA1	Annexin A1: regulator of the inflammatory process as an effector of glucocorticoid-mediated responses
	down	PIK3CG	Phosphatidylinositol 4,5-bisphosphate 3-kinase catalytic subunit gamma isoform: kinase involved in generation of phosphatidylinositol 3,4,5-triphosphate (PIP3)
	down	TNS1	Tensin-1: involved in fibrillar adhesion formation
CBE-YE1 H2-gRNA vs. CBE-E63A-YE1 H2-gRNA	up	CU633904.1	Long intergenic non-coding RNA; function unknown
	down	HBG2	Hemoglobin subunit gamma-2: gamma chain of fetal hemoglobin

Supplementary Figure 13. Hits from differential expression analysis of HEK293T cells undergoing base editing at the *HEK2* (H2) genomic locus by RNA off-target optimized constructs. HEK293T cells were transfected with the reduced-RNA editing variants ABE-F148A or CBE-YE1, or their catalytically inactivated deaminase counterparts ABE-E59A-F148A or CBE-E63A-YE1, CBEΔUGI-E63A-YE1 and either a HEK2-targeting gRNA (H2-gRNA) or a non-targeting gRNA (nt-gRNA). At 48 hours post-transfection, cells were lysed, the RNA was extracted, and the coding transcriptome of the RNA libraries was enriched and subjected to HTS. Differential expression analyses were performed based on the negative binomial distribution using the *DESeq2* package. Listed are transcripts found to be up- or down-regulated in the various comparisons indicated (ABE-F148A H2-gRNA vs. ABE-F148A nt-gRNA, ABE-F148A H2-gRNA vs ABE-E59A-F148A H2-gRNA, CBE-YE1 H2-gRNA vs. CBE-YE1 nt-gRNA, and CBE-YE1 H2-gRNA vs. CBE-E63A-YE1 H2-gRNA), and their function. Statistically significantly differentially expressed genes were defined as genes where the absolute value of the $\log_2$(fold change)>1 and the adjusted p-value≤0.01.

Supplementary Sequences

Protein Sequence of ABE7.10-P2A-GFP (TadA-TadA*-nCas9-NLS):
MKRTADGSEFESPKKKRKVSEVEFSHEYWMRHALTLAKRAWDEREVPVGAVLVHNNRVIGEGWNRPIGRHD
PTAHAEIMALRQGGLVMQNYRLIDATLYVTLEPCVMCAGAMIHSRIGRVVFGARDAKTGAAGSLMDVLHHPGM
NHRVEITEGILADECAALLSDFFRMRRQEIKAQKKAQSSTDSGGSSGGSSGSETPGTSESATPESSGGSSGGS
SEVEFSHEYWMRHALTLAKRARDEREVPVGAVLVLNNRVIGEGWNRAIGLHDPTAHAEIMALRQGGLVMQNY
RLIDATLYVTFEPCVMCAGAMIHSRIGRVVFGVRNAKTGAAGSLMDVLHYPGMNHRVEITEGILADECAALLCY
FFRMPRQVFNAQKKAQSSTDSGGSSGGSSGSETPGTSESATPESSGGSSGGSDKKYSIGLAIGTNSVGWAVI
TDEYKVPSKKFKVLGNTDRHSIKKNLIGALLFDSGETAEATRLKRTARRRYTRRKNRICYLQEIFSNEMAKVDDS
FFHRLEESFLVEEDKKHERHPIFGNIVDEVAYHEKYPTIYHLRKKLVDSTDKADLRLIYLALAHMIKFRGHFLIEG
DLNPDNSDVDKLFIQLVQTYNQLFEENPINASGVDAKAILSARLSKSRRLENLIAQLPGEKKNGLFGNLIALSLGL
TPNFKSNFDLAEDAKLQLSKDTYDDDLDNLLAQIGDQYADLFLAAKNLSDAILLSDILRVNTEITKAPLSASMIKR
YDEHHQDLTLLKALVRQQLPEKYKEIFFDQSKNGYAGYIDGGASQEEFYKFIKPILEKMDGTEELLVKLNREDLL
RKQRTFDNGSIPHQIHLGELHAILRRQEDFYPFLKDNREKIEKILTFRIPYYVGPLARGNSRFAWMTRKSEETITP
WNFEEVVDKGASAQSFIERMTNFDKNLPNEKVLPKHSLLYEYFTVYNELTKVKYVTEGMRKPAFLSGEQKKAI
VDLLFKTNRKVTVKQLKEDYFKKIECFDSVEISGVEDRFNASLGTYHDLLKIIKDKDFLDNEENEDILEDIVLTLTL
FEDREMIEERLKTYAHLFDDKVMKQLKRRRYTGWGRLSRKLINGIRDKQSGKTILDFLKSDGFANRNFMQLIHD
DSLTFKEDIQKAQVSGQGDSLHEHIANLAGSPAIKKGILQTVKVVDELVKVMGRHKPENIVIEMARENQTTQKG
QKNSRERMKRIEEGIKELGSQILKEHPVENTQLQNEKLYLYYLQNGRDMYVDQELDINRLSDYDVDHIVPQSFL
KDDSIDNKVLTRSDKNRGKSDNVPSEEVVKKMKNYWRQLLNAKLITQRKFDNLTKAERGGLSELDKAGFIKRQ
LVETRQITKHVAQILDSRMNTKYDENDKLIREVKVITLKSKLVSDFRKDFQFYKVREINNYHHAHDAYLNAVVGT
ALIKKYPKLESEFVYGDYKVYDVRKMIAKSEQEIGKATAKYFFYSNIMNFFKTEITLANGEIRKRPLIETNGETGEI
VWDKGRDFATVRKVLSMPQVNIVKKTEVQTGGFSKESILPKRNSDKLIARKKDWDPKKYGGFDSPTVAYSVLV
VAKVEKGKSKKLKSVKELLGITIMERSSFEKNPIDFLEAKGYKEVKKDLIIKLPKYSLFELENGRKRMLASAGELQ
KGNELALPSKYVNFLYLASHYEKLKGSPEDNEQKQLFVEQHKHYLDEIIEQISEFSKRVILADANLDKVLSAYNK
HRDKPIREQAENIIHLFTLTNLGAPAAFKYFDTTIDRKRYTSTKEVLDATLIHQSITGLYETRIDLSQLGGDSGGSK
RTADGSEFEPKKKRKVDSGGSKRTADGSEFEPKKKRKVGSGATNFSLLKQAGDVEENPGPMVSKGEELFTG
VVPILVELDGDVNGHKFSVSGEGEGDATYGKLTLKFICTTGKLPVPWPTLVTTLTYGVQCFSRYPDHMKQHDF
FKSAMPEGYVQERTIFFKDDGNYKTRAEVKFEGDTLVNRIELKGIDFKEDGNILGHKLEYNYNSHNVYIMADKQ
KNGIKVNFKIRHNIEDGSVQLADHYQQNTPIGDGPVLLPDNHYLSTQSALSKDPNEKRDHMVLLEFVTAAGITL
GMDELYKSGGSPKKKRKV

Protein Sequence of CBE: (BE4-ancAPOBEC1-P2A-GFP)

MKRTADGSEFESPKKKRKVSSETGPVAVDPTLRRRIEPHEFEVFFDPRELRKETCLLYEIKWGTSHKIWRHSS
KNTTKHVEVNFIEKFTSERHFCPSTSCSITWFLSWSPCGECSKAITEFLSQHPNVTLVIYVARLYHHMDQQNRQ
GLRDLVNSGVTIQIMTAPEYDYCWRNFVNYPPGKEAHWPRYPPLWMKLYALELHAGILGLPPCLNILRRKQPQ
LTFFTIALQSCHYQRLPPHILWATGLKSGGSSGGSSGSETPGTSESATPESSGGSSGGSDKKYSIGLAIGTNSV
GWAVITDEYKVPSKKFKVLGNTDRHSIKKNLIGALLFDSGETAEATRLKRTARRRYTRRKNRICYLQEIFSNEMA
KVDDSFFHRLEESFLVEEDKKHERHPIFGNIVDEVAYHEKYPTIYHLRKKLVDSTDKADLRLIYLALAHMIKFRGH
FLIEGDLNPDNSDVDKLFIQLVQTYNQLFEENPINASGVDAKAILSARLSKSRRLENLIAQLPGEKKNGLFGNLIA
LSLGLTPNFKSNFDLAEDAKLQLSKDTYDDDLDNLLAQIGDQYADLFLAAKNLSDAILLSDILRVNTEITKAPLSA
SMIKRYDEHHQDLTLLKALVRQQLPEKYKEIFFDQSKNGYAGYIDGGASQEEFYKFIKPILEKMDGTEELLVKLN
REDLLRKQRTFDNGSIPHQIHLGELHAILRRQEDFYPFLKDNREKIEKILTFRIPYYVGPLARGNSRFAWMTRKS
EETITPWNFEEVVDKGASAQSFIERMTNFDKNLPNEKVLPKHSLLYEYFTVYNELTKVKYVTEGMRKPAFLSGE
QKKAIVDLLFKTNRKVTVKQLKEDYFKKIECFDSVEISGVEDRFNASLGTYHDLLKIIKDKDFLDNEENEDILEDIV
LTLTLFEDREMIEERLKTYAHLFDDKVMKQLKRRRYTGWGRLSRKLINGIRDKQSGKTILDFLKSDGFANRNFM
QLIHDDSLTFKEDIQKAQVSGQGDSLHEHIANLAGSPAIKKGILQTVKVVDELVKVMGRHKPENIVIEMARENQT
TQKGQKNSRERMKRIEEGIKELGSQILKEHPVENTQLQNEKLYLYYLQNGRDMYVDQELDINRLSDYDVDHIVP
QSFLKDDSIDNKVLTRSDKNRGKSDNVPSEEVVKKMKNYWRQLLNAKLITQRKFDNLTKAERGGLSELDKAGF

IKRQLVETRQITKHVAQILDSRMNTKYDENDKLIREVKVITLKSKLVSDFRKDFQFYKVREINNYHHAHDAYLNAV
VGTALIKKYPKLESEFVYGDYKVYDVRKMIAKSEQEIGKATAKYFFYSNIMNFFKTEITLANGEIRKRPLIETNGET
GEIVWDKGRDFATVRKVLSMPQVNIVKKTEVQTGGFSKESILPKRNSDKLIARKKDWDPKKYGGFDSPTVAYS
VLVVAKVEKGKSKKLKSVKELLGITIMERSSFEKNPIDFLEAKGYKEVKKDLIIKLPKYSLFELENGRKRMLASAG
ELQKGNELALPSKYVNFLYLASHYEKLKGSPEDNEQKQLFVEQHKHYLDEIIEQISEFSKRVILADANLDKVLSA
YNKHRDKPIREQAENIIHLFTLTNLGAPAAFKYFDTTIDRKRYTSTKEVLDATLIHQSITGLYETRIDLSQLGGDSG
GSGGSGGSTNLSDIIEKETGKQLVIQESILMLPEEVEEVIGNKPESDILVHTAYDESTDENVMLLTSDAPEYKPW
ALVIQDSNGENKIKMLSGGSGGSGGSTNLSDIIEKETGKQLVIQESILMLPEEVEEVIGNKPESDILVHTAYDEST
DENVMLLTSDAPEYKPWALVIQDSNGENKIKMLSGGSKRTADGSEFEPKKKRKVGSGATNFSLLKQAGDVEE
NPGPMVSKGEELFTGVVPILVELDGDVNGHKFSVSGEGEGDATYGKLTLKFICTTGKLPVPWPTLVTTLTYG
VQCFSRYPDHMKQHDFFKSAMPEGYVQERTIFFKDDGNYKTRAEVKFEGDTLVNRIELKGIDFKEDGNILGH
KLEYNYNSHNVYIMADKQKNGIKVNFKIRHNIEDGSVQLADHYQQNTPIGDGPVLLPDNHYLSTQSALSKDPN
EKRDHMVLLEFVTAAGITLGMDELYKSGGSPKKKRKV

Protein Sequence of CBEΔUGI:

MKRTADGSEFESPKKKRKVSSETGPVAVDPTLRRRIEPHEFEVFFDPRELRKETCLLYEIKWGTSHKIWRHSS
KNTTKHVEVNFIEKFTSERHFCPSTSCSITWFLSWSPCGECSKAITEFLSQHPNVTLVIYVARLYHHMDQQNRQ
GLRDLVNSGVTIQIMTAPEYDYCWRNFVNYPPGKEAHWPRYPPLWMKLYALELHAGILGLPPCLNILRRKQPQ
LTFFTIALQSCHYQRLPPHILWATGLKSGGSSGGSSGSETPGTSESATPESSGGSSGGSDKKYSIGLAIGTNSV
GWAVITDEYKVPSKKFKVLGNTDRHSIKKNLIGALLFDSGETAEATRLKRTARRRYTRRKNRICYLQEIFSNEMA
KVDDSFFHRLEESFLVEEDKKHERHPIFGNIVDEVAYHEKYPTIYHLRKKLVDSTDKADLRLIYLALAHMIKFRGH
FLIEGDLNPDNSDVDKLFIQLVQTYNQLFEENPINASGVDAKAILSARLSKSRRLENLIAQLPGEKKNGLFGNLIA
LSLGLTPNFKSNFDLAEDAKLQLSKDTYDDDLDNLLAQIGDQYADLFLAAKNLSDAILLSDILRVNTEITKAPLSA
SMIKRYDEHHQDLTLLKALVRQQLPEKYKEIFFDQSKNGYAGYIDGGASQEEFYKFIKPILEKMDGTEELLVKLN
REDLLRKQRTFDNGSIPHQIHLGELHAILRRQEDFYPFLKDNREKIEKILTFRIPYYVGPLARGNSRFAWMTRKS
EETITPWNFEEVVDKGASAQSFIERMTNFDKNLPNEKVLPKHSLLYEYFTVYNELTKVKYVTEGMRKPAFLSGE
QKKAIVDLLFKTNRKVTVKQLKEDYFKKIECFDSVEISGVEDRFNASLGTYHDLLKIIKDKDFLDNEENEDILEDIV
LTLTLFEDREMIEERLKTYAHLFDDKVMKQLKRRRYTGWGRLSRKLINGIRDKQSGKTILDFLKSDGFANRNFM
QLIHDDSLTFKEDIQKAQVSGQGDSLHEHIANLAGSPAIKKGILQTVKVVDELVKVMGRHKPENIVIEMARENQT
TQKGQKNSRERMKRIEEGIKELGSQILKEHPVENTQLQNEKLYLYYLQNGRDMYVDQELDINRLSDYDVDHIVP
QSFLKDDSIDNKVLTRSDKNRGKSDNVPSEEVVKKMKNYWRQLLNAKLITQRKFDNLTKAERGGLSELDKAGF
IKRQLVETRQITKHVAQILDSRMNTKYDENDKLIREVKVITLKSKLVSDFRKDFQFYKVREINNYHHAHDAYLNAV
VGTALIKKYPKLESEFVYGDYKVYDVRKMIAKSEQEIGKATAKYFFYSNIMNFFKTEITLANGEIRKRPLIETNGET
GEIVWDKGRDFATVRKVLSMPQVNIVKKTEVQTGGFSKESILPKRNSDKLIARKKDWDPKKYGGFDSPTVAYS
VLVVAKVEKGKSKKLKSVKELLGITIMERSSFEKNPIDFLEAKGYKEVKKDLIIKLPKYSLFELENGRKRMLASAG
ELQKGNELALPSKYVNFLYLASHYEKLKGSPEDNEQKQLFVEQHKHYLDEIIEQISEFSKRVILADANLDKVLSA
YNKHRDKPIREQAENIIHLFTLTNLGAPAAFKYFDTTIDRKRYTSTKEVLDATLIHQSITGLYETRIDLSQLGGDKR
TADGSEFEPKKKRKVGSGATNFSLLKQAGDVEENPGPMVSKGEELFTGVVPILVELDGDVNGHKFSVSGEGE
GDATYGKLTLKFICTTGKLPVPWPTLVTTLTYGVQCFSRYPDHMKQHDFFKSAMPEGYVQERTIFFKDDGNYK
TRAEVKFEGDTLVNRIELKGIDFKEDGNILGHKLEYNYNSHNVYIMADKQKNGIKVNFKIRHNIEDGSVQLADHY
QQNTPIGDGPVLLPDNHYLSTQSALSKDPNEKRDHMVLLEFVTAAGITLGMDELYKSGGSPKKKRKV

Protein Sequence of ABE(dCas9):

MKRTADGSEFESPKKKRKVSEVEFSHEYWMRHALTLAKRAWDEREVPVGAVLVHNNRVIGEGWNRPIGRHD
PTAHAEIMALRQGGLVMQNYRLIDATLYVTLEPCVMCAGAMIHSRIGRVVFGARDAKTGAAGSLMDVLHHPGM
NHRVEITEGILADECAALLSDFFRMRRQEIKAQKKAQSSTDSGGSSGGSSGSETPGTSESATPESSGGSSGGS
SEVEFSHEYWMRHALTLAKRARDEREVPVGAVLVLNNRVIGEGWNRAIGLHDPTAHAEIMALRQGGLVMQNY
RLIDATLYVTFEPCVMCAGAMIHSRIGRVVFGVRNAKTGAAGSLMDVLHYPGMNHRVEITEGILADECAALLCY

FFRMPRQVFNAQKKAQSSTDSGGSSGGSSGSETPGTSESATPESSGGSSGGSDKKYSIGLAIGTNSVGWAVI
TDEYKVPSKKFKVLGNTDRHSIKKNLIGALLFDSGETAEATRLKRTARRRYTRRKNRICYLQEIFSNEMAKVDDS
FFHRLEESFLVEEDKKHERHPIFGNIVDEVAYHEKYPTIYHLRKKLVDSTDKADLRLIYLALAHMIKFRGHFLIEG
DLNPDNSDVDKLFIQLVQTYNQLFEENPINASGVDAKAILSARLSKSRRLENLIAQLPGEKKNGLFGNLIALSLGL
TPNFKSNFDLAEDAKLQLSKDTYDDDLDNLLAQIGDQYADLFLAAKNLSDAILLSDILRVNTEITKAPLSASMIKR
YDEHHQDLTLLKALVRQQLPEKYKEIFFDQSKNGYAGYIDGGASQEEFYKFIKPILEKMDGTEELLVKLNREDLL
RKQRTFDNGSIPHQIHLGELHAILRRQEDFYPFLKDNREKIEKILTFRIPYYVGPLARGNSRFAWMTRKSEETITP
WNFEEVVDKGASAQSFIERMTNFDKNLPNEKVLPKHSLLYEYFTVYNELTKVKYVTEGMRKPAFLSGEQKKAI
VDLLFKTNRKVTVKQLKEDYFKKIECFDSVEISGVEDRFNASLGTYHDLLKIIKDKDFLDNEENEDILEDIVLTLTL
FEDREMIEERLKTYAHLFDDKVMKQLKRRRYTGWGRLSRKLINGIRDKQSGKTILDFLKSDGFANRNFMQLIHD
DSLTFKEDIQKAQVSGQGDSLHEHIANLAGSPAIKKGILQTVKVVDELVKVMGRHKPENIVIEMARENQTTQKG
QKNSRERMKRIEEGIKELGSQILKEHPVENTQLQNEKLYLYYLQNGRDMYVDQELDINRLSDYDVDAIVPQSFL
KDDSIDNKVLTRSDKNRGKSDNVPSEEVVKKMKNYWRQLLNAKLITQRKFDNLTKAERGGLSELDKAGFIKRQ
LVETRQITKHVAQILDSRMNTKYDENDKLIREVKVITLKSKLVSDFRKDFQFYKVREINNYHHAHDAYLNAVVGT
ALIKKYPKLESEFVYGDYKVYDVRKMIAKSEQEIGKATAKYFFYSNIMNFFKTEITLANGEIRKRPLIETNGETGEI
VWDKGRDFATVRKVLSMPQVNIVKKTEVQTGGFSKESILPKRNSDKLIARKKDWDPKKYGGFDSPTVAYSVLV
VAKVEKGKSKKLKSVKELLGITIMERSSFEKNPIDFLEAKGYKEVKKDLIIKLPKYSLFELENGRKRMLASAGELQ
KGNELALPSKYVNFLYLASHYEKLKGSPEDNEQKQLFVEQHKHYLDEIIEQISEFSKRVILADANLDKVLSAYNK
HRDKPIREQAENIIHLFTLTNLGAPAAFKYFDTTIDRKRYTSTKEVLDATLIHQSITGLYETRIDLSQLGGDSGGSK
RTADGSEFEPKKKRKVGSGATNFSLLKQAGDVEENPGPMVSKGEELFTGVVPILVELDGDVNGHKFSVSGEG
EGDATYGKLTLKFICTTGKLPVPWPTLVTTLTYGVQCFSRYPDHMKQHDFFKSAMPEGYVQERTIFFKDDGNY
KTRAEVKFEGDTLVNRIELKGIDFKEDGNILGHKLEYNYNSHNVYIMADKQKNGIKVNFKIRHNIEDGSVQLADH
YQQNTPIGDGPVLLPDNHYLSTQSALSKDPNEKRDHMVLLEFVTAAGITLGMDELYKSGGSPKKKRKV

Protein Sequence of CBE(dCas9):

MKRTADGSEFESPKKKRKVSSETGPVAVDPTLRRRIEPHEFEVFFDPRELRKETCLLYEIKWGTSHKIWRHSS
KNTTKHVEVNFIEKFTSERHFCPSTSCSITWFLSWSPCGECSKAITEFLSQHPNVTLVIYVARLYHHMDQQNRQ
GLRDLVNSGVTIQIMTAPEYDYCWRNFVNYPPGKEAHWPRYPPLWMKLYALELHAGILGLPPCLNILRRKQPQ
LTFFTIALQSCHYQRLPPHILWATGLKSGGSSGGSSGSETPGTSESATPESSGGSSGGSDKKYSIGLAIGTNSV
GWAVITDEYKVPSKKFKVLGNTDRHSIKKNLIGALLFDSGETAEATRLKRTARRRYTRRKNRICYLQEIFSNEMA
KVDDSFFHRLEESFLVEEDKKHERHPIFGNIVDEVAYHEKYPTIYHLRKKLVDSTDKADLRLIYLALAHMIKFRGH
FLIEGDLNPDNSDVDKLFIQLVQTYNQLFEENPINASGVDAKAILSARLSKSRRLENLIAQLPGEKKNGLFGNLIA
LSLGLTPNFKSNFDLAEDAKLQLSKDTYDDDLDNLLAQIGDQYADLFLAAKNLSDAILLSDILRVNTEITKAPLSA
SMIKRYDEHHQDLTLLKALVRQQLPEKYKEIFFDQSKNGYAGYIDGGASQEEFYKFIKPILEKMDGTEELLVKLN
REDLLRKQRTFDNGSIPHQIHLGELHAILRRQEDFYPFLKDNREKIEKILTFRIPYYVGPLARGNSRFAWMTRKS
EETITPWNFEEVVDKGASAQSFIERMTNFDKNLPNEKVLPKHSLLYEYFTVYNELTKVKYVTEGMRKPAFLSGE
QKKAIVDLLFKTNRKVTVKQLKEDYFKKIECFDSVEISGVEDRFNASLGTYHDLLKIIKDKDFLDNEENEDILEDIV
LTLTLFEDREMIEERLKTYAHLFDDKVMKQLKRRRYTGWGRLSRKLINGIRDKQSGKTILDFLKSDGFANRNFM
QLIHDDSLTFKEDIQKAQVSGQGDSLHEHIANLAGSPAIKKGILQTVKVVDELVKVMGRHKPENIVIEMARENQT
TQKGQKNSRERMKRIEEGIKELGSQILKEHPVENTQLQNEKLYLYYLQNGRDMYVDQELDINRLSDYDVDAIVP
QSFLKDDSIDNKVLTRSDKNRGKSDNVPSEEVVKKMKNYWRQLLNAKLITQRKFDNLTKAERGGLSELDKAGF
IKRQLVETRQITKHVAQILDSRMNTKYDENDKLIREVKVITLKSKLVSDFRKDFQFYKVREINNYHHAHDAYLNAV
VGTALIKKYPKLESEFVYGDYKVYDVRKMIAKSEQEIGKATAKYFFYSNIMNFFKTEITLANGEIRKRPLIETNGET
GEIVWDKGRDFATVRKVLSMPQVNIVKKTEVQTGGFSKESILPKRNSDKLIARKKDWDPKKYGGFDSPTVAYS
VLVVAKVEKGKSKKLKSVKELLGITIMERSSFEKNPIDFLEAKGYKEVKKDLIIKLPKYSLFELENGRKRMLASAG
ELQKGNELALPSKYVNFLYLASHYEKLKGSPEDNEQKQLFVEQHKHYLDEIIEQISEFSKRVILADANLDKVLSA
YNKHRDKPIREQAENIIHLFTLTNLGAPAAFKYFDTTIDRKRYTSTKEVLDATLIHQSITGLYETRIDLSQLGGDSG
GSGGSGGSTNLSDIIEKETGKQLVIQESILMLPEEVEEVIGNKPESDILVHTAYDESTDENVMLLTSDAPEYKPW

ALVIQDSNGENKIKMLSGGSGGSGGSTNLSDIIEKETGKQLVIQESILMLPEEVEEVIGNKPESDILVHTAYDEST
DENVMLLTSDAPEYKPWALVIQDSNGENKIKMLSGGSKRTADGSEFEPKKKRKVGSGATNFSLLKQAGDVEE
NPGP**MVSKGEELFTGVVPILVELDGDVNGHKFSVSGEGEGDATYGKLTLKFICTTGKLPVPWPTLVTTLTYG**
VQCFSRYPDHMKQHDFFKSAMPEGYVQERTIFFKDDGNYKTRAEVKFEGDTLVNRIELKGIDFKEDGNILGH
KLEYNYNSHNVYIMADKQKNGIKVNFKIRHNIEDGSVQLADHYQQNTPIGDGPVLLPDNHYLSTQSALSKDPN
EKRDHMVLLEFVTAAGITLGMDELYK

Protein Sequence of CBEΔUGI(dCas9):

MKRTADGSEFESPKKKRKVSSETGPVAVDPTLRRRIEPHEFEVFFDPRELRKETCLLYEIKWGTSHKIWRHSS
KNTTKHVEVNFIEKFTSERHFCPSTSCSITWFLSWSPCGECSKAITEFLSQHPNVTLVIYVARLYHHMDQQNRQ
GLRDLVNSGVTIQIMTAPEYDYCWRNFVNYPPGKEAHWPRYPPLWMKLYALELHAGILGLPPCLNILRRKQPQ
LTFFTIALQSCHYQRLPPHILWATGLKSGGSSGGSSGSETPGTSESATPESSGGSSGGSDKKYSIGLAIGTNSV
GWAVITDEYKVPSKKFKVLGNTDRHSIKKNLIGALLFDSGETAEATRLKRTARRRYTRRKNRICYLQEIFSNEMA
KVDDSFFHRLEESFLVEEDKKHERHPIFGNIVDEVAYHEKYPTIYHLRKKLVDSTDKADLRLIYLALAHMIKFRGH
FLIEGDLNPDNSDVDKLFIQLVQTYNQLFEENPINASGVDAKAILSARLSKSRRLENLIAQLPGEKKNGLFGNLIA
LSLGLTPNFKSNFDLAEDAKLQLSKDTYDDDLDNLLAQIGDQYADLFLAAKNLSDAILLSDILRVNTEITKAPLSA
SMIKRYDEHHQDLTLLKALVRQQLPEKYKEIFFDQSKNGYAGYIDGGASQEEFYKFIKPILEKMDGTEELLVKLN
REDLLRKQRTFDNGSIPHQIHLGELHAILRRQEDFYPFLKDNREKIEKILTFRIPYYVGPLARGNSRFAWMTRKS
EETITPWNFEEVVDKGASAQSFIERMTNFDKNLPNEKVLPKHSLLYEYFTVYNELTKVKYVTEGMRKPAFLSGE
QKKAIVDLLFKTNRKVTVKQLKEDYFKKIECFDSVEISGVEDRFNASLGTYHDLLKIIKDKDFLDNEENEDILEDIV
LTLTLFEDREMIEERLKTYAHLFDDKVMKQLKRRRYTGWGRLSRKLINGIRDKQSGKTILDFLKSDGFANRNFM
QLIHDDSLTFKEDIQKAQVSGQGDSLHEHIANLAGSPAIKKGILQTVKVVDELVKVMGRHKPENIVIEMARENQT
TQKGQKNSRERMKRIEEGIKELGSQILKEHPVENTQLQNEKLYLYYLQNGRDMYVDQELDINRLSDYDVDAIVP
QSFLKDDSIDNKVLTRSDKNRGKSDNVPSEEVVKKMKNYWRQLLNAKLITQRKFDNLTKAERGGLSELDKAGF
IKRQLVETRQITKHVAQILDSRMNTKYDENDKLIREVKVITLKSKLVSDFRKDFQFYKVREINNYHHAHDAYLNAV
VGTALIKKYPKLESEFVYGDYKVYDVRKMIAKSEQEIGKATAKYFFYSNIMNFFKTEITLANGEIRKRPLIETNGET
GEIVWDKGRDFATVRKVLSMPQVNIVKKTEVQTGGFSKESILPKRNSDKLIARKKDWDPKKYGGFDSPTVAYS
VLVVAKVEKGKSKKLKSVKELLGITIMERSSFEKNPIDFLEAKGYKEVKKDLIIKLPKYSLFELENGRKRMLASAG
ELQKGNELALPSKYVNFLYLASHYEKLKGSPEDNEQKQLFVEQHKHYLDEIIEQISEFSKRVILADANLDKVLSA
YNKHRDKPIREQAENIIHLFTLTNLGAPAAFKYFDTTIDRKRYTSTKEVLDATLIHQSITGLYETRIDLSQLGGDKR
TADGSEFEPKKKRKVGSGATNFSLLKQAGDVEENPGPMVSKGEELFTGVVPILVELDGDVNGHKFSVSGEGE
GDATYGKLTLKFICTTGKLPVPWPTLVTTLTYGVQCFSRYPDHMKQHDFFKSAMPEGYVQERTIFFKDDGNYK
TRAEVKFEGDTLVNRIELKGIDFKEDGNILGHKLEYNYNSHNVYIMADKQKNGIKVNFKIRHNIEDGSVQLADHY
QQNTPIGDGPVLLPDNHYLSTQSALSKDPNEKRDHMVLLEFVTAAGITLGMDELYKSGGSPKKKRKV*PVIITIT

Protein Sequence of ABE-F148A:

MKRTADGSEFESPKKKRKVSEVEFSHEYWMRHALTLAKRAWDEREVPVGAVLVHNNRVIGEGWNRPIGRHD
PTAHAEIMALRQGGLVMQNYRLIDATLYVTLEPCVMCAGAMIHSRIGRVVFGARDAKTGAAGSLMDVLHHPGM
NHRVEITEGILADECAALLSDAFRMRRQEIKAQKKAQSSTDSGGSSGGSSGSETPGTSESATPESSGGSSGG
SSEVEFSHEYWMRHALTLAKRARDEREVPVGAVLVLNNRVIGEGWNRAIGLHDPTAHAEIMALRQGGLVMQN
YRLIDATLYVTFEPCVMCAGAMIHSRIGRVVFGVRNAKTGAAGSLMDVLHYPGMNHRVEITEGILADECAALLC
YAFRMPRQVFNAQKKAQSSTDSGGSSGGSSGSETPGTSESATPESSGGSSGGSDKKYSIGLAIGTNSVGWA
VITDEYKVPSKKFKVLGNTDRHSIKKNLIGALLFDSGETAEATRLKRTARRRYTRRKNRICYLQEIFSNEMAKVD
DSFFHRLEESFLVEEDKKHERHPIFGNIVDEVAYHEKYPTIYHLRKKLVDSTDKADLRLIYLALAHMIKFRGHFLIE
GDLNPDNSDVDKLFIQLVQTYNQLFEENPINASGVDAKAILSARLSKSRRLENLIAQLPGEKKNGLFGNLIALSLG

LTPNFKSNFDLAEDAKLQLSKDTYDDDLDNLLAQIGDQYADLFLAAKNLSDAILLSDILRVNTEITKAPLSASMIKR
YDEHHQDLTLLKALVRQQLPEKYKEIFFDQSKNGYAGYIDGGASQEEFYKFIKPILEKMDGTEELLVKLNREDLL
RKQRTFDNGSIPHQIHLGELHAILRRQEDFYPFLKDNREKIEKILTFRIPYYVGPLARGNSRFAWMTRKSEETITP
WNFEEVVDKGASAQSFIERMTNFDKNLPNEKVLPKHSLLYEYFTVYNELTKVKYVTEGMRKPAFLSGEQKKAI
VDLLFKTNRKVTVKQLKEDYFKKIECFDSVEISGVEDRFNASLGTYHDLLKIIKDKDFLDNEENEDILEDIVLTLTL
FEDREMIEERLKTYAHLFDDKVMKQLKRRRYTGWGRLSRKLINGIRDKQSGKTILDFLKSDGFANRNFMQLIHD
DSLTFKEDIQKAQVSGQGDSLHEHIANLAGSPAIKKGILQTVKVVDELVKVMGRHKPENIVIEMARENQTTQKG
QKNSRERMKRIEEGIKELGSQILKEHPVENTQLQNEKLYLYYLQNGRDMYVDQELDINRLSDYDVDHIVPQSFL
KDDSIDNKVLTRSDKNRGKSDNVPSEEVVKKMKNYWRQLLNAKLITQRKFDNLTKAERGGLSELDKAGFIKRQ
LVETRQITKHVAQILDSRMNTKYDENDKLIREVKVITLKSKLVSDFRKDFQFYKVREINNYHHAHDAYLNAVVGT
ALIKKYPKLESEFVYGDYKVYDVRKMIAKSEQEIGKATAKYFFYSNIMNFFKTEITLANGEIRKRPLIETNGETGEI
VWDKGRDFATVRKVLSMPQVNIVKKTEVQTGGFSKESILPKRNSDKLIARKKDWDPKKYGGFDSPTVAYSVLV
VAKVEKGKSKKLKSVKELLGITIMERSSFEKNPIDFLEAKGYKEVKKDLIIKLPKYSLFELENGRKRMLASAGELQ
KGNELALPSKYVNFLYLASHYEKLKGSPEDNEQKQLFVEQHKHYLDEIIEQISEFSKRVILADANLDKVLSAYNK
HRDKPIREQAENIIHLFTLTNLGAPAAFKYFDTTIDRKRYTSTKEVLDATLIHQSITGLYETRIDLSQLGGDSGGSK
RTADGSEFEPKKKRKVGSGATNFSLLKQAGDVEENPGPMVSKGEELFTGVVPILVELDGDVNGHKFSVSGEG
EGDATYGKLTLKFICTTGKLPVPWPTLVTTLTYGVQCFSRYPDHMKQHDFFKSAMPEGYVQERTIFFKDDGNY
KTRAEVKFEGDTLVNRIELKGIDFKEDGNILGHKLEYNYNSHNVYIMADKQKNGIKVNFKIRHNIEDGSVQLADH
YQQNTPIGDGPVLLPDNHYLSTQSALSKDPNEKRDHMVLLEFVTAAGITLGMDELYKSGGSPKKKRKV

Protein Sequence of CBE-YE1:

MKRTADGSEFESPKKKRKVSSETGPVAVDPTLRRRIEPHEFEVFFDPRELRKETCLLYEINWGGRHSIWRHTS
QNTNKHVEVNFIEKFTTERYFCPNTRCSITWFLSYSPCGECSRAITEFLSRYPHVTLFIYIARLYHHADPENRQG
LRDLISSGVTIQIMTEQESGYCWRNFVNYSPSNEAHWPRYPHLWVRLYVLELYCIILGLPPCLNILRRKQPQLTF
FTIALQSCHYQRLPPHILWATGLKSGGSSGGSSGSETPGTSESATPESSGGSSGGSDKKYSIGLAIGTNSVGW
AVITDEYKVPSKKFKVLGNTDRHSIKKNLIGALLFDSGETAEATRLKRTARRRYTRRKNRICYLQEIFSNEMAKV
DDSFFHRLEESFLVEEDKKHERHPIFGNIVDEVAYHEKYPTIYHLRKKLVDSTDKADLRLIYLALAHMIKFRGHFLI
EGDLNPDNSDVDKLFIQLVQTYNQLFEENPINASGVDAKAILSARLSKSRRLENLIAQLPGEKKNGLFGNLIALSL
GLTPNFKSNFDLAEDAKLQLSKDTYDDDLDNLLAQIGDQYADLFLAAKNLSDAILLSDILRVNTEITKAPLSASMI
KRYDEHHQDLTLLKALVRQQLPEKYKEIFFDQSKNGYAGYIDGGASQEEFYKFIKPILEKMDGTEELLVKLNRE
DLLRKQRTFDNGSIPHQIHLGELHAILRRQEDFYPFLKDNREKIEKILTFRIPYYVGPLARGNSRFAWMTRKSEE
TITPWNFEEVVDKGASAQSFIERMTNFDKNLPNEKVLPKHSLLYEYFTVYNELTKVKYVTEGMRKPAFLSGEQK
KAIVDLLFKTNRKVTVKQLKEDYFKKIECFDSVEISGVEDRFNASLGTYHDLLKIIKDKDFLDNEENEDILEDIVLTL
TLFEDREMIEERLKTYAHLFDDKVMKQLKRRRYTGWGRLSRKLINGIRDKQSGKTILDFLKSDGFANRNFMQLI
HDDSLTFKEDIQKAQVSGQGDSLHEHIANLAGSPAIKKGILQTVKVVDELVKVMGRHKPENIVIEMARENQTTQ
KGQKNSRERMKRIEEGIKELGSQILKEHPVENTQLQNEKLYLYYLQNGRDMYVDQELDINRLSDYDVDHIVPQS
FLKDDSIDNKVLTRSDKNRGKSDNVPSEEVVKKMKNYWRQLLNAKLITQRKFDNLTKAERGGLSELDKAGFIK
RQLVETRQITKHVAQILDSRMNTKYDENDKLIREVKVITLKSKLVSDFRKDFQFYKVREINNYHHAHDAYLNAVV
GTALIKKYPKLESEFVYGDYKVYDVRKMIAKSEQEIGKATAKYFFYSNIMNFFKTEITLANGEIRKRPLIETNGET
GEIVWDKGRDFATVRKVLSMPQVNIVKKTEVQTGGFSKESILPKRNSDKLIARKKDWDPKKYGGFDSPTVAYS
VLVVAKVEKGKSKKLKSVKELLGITIMERSSFEKNPIDFLEAKGYKEVKKDLIIKLPKYSLFELENGRKRMLASAG
ELQKGNELALPSKYVNFLYLASHYEKLKGSPEDNEQKQLFVEQHKHYLDEIIEQISEFSKRVILADANLDKVLSA
YNKHRDKPIREQAENIIHLFTLTNLGAPAAFKYFDTTIDRKRYTSTKEVLDATLIHQSITGLYETRIDLSQLGGDSG
GSGGSGGSTNLSDIIEKETGKQLVIQESILMLPEEVEEVIGNKPESDILVHTAYDESTDENVMLLTSDAPEYKPW
ALVIQDSNGENKIKMLSGGSGGSGGSTNLSDIIEKETGKQLVIQESILMLPEEVEEVIGNKPESDILVHTAYDEST
DENVMLLTSDAPEYKPWALVIQDSNGENKIKMLSGGSKRTADGSEFEPKKKRKVGSGATNFSLLKQAGDVEE
NPGPMVSKGEELFTGVVPILVELDGDVNGHKFSVSGEGEGDATYGKLTLKFICTTGKLPVPWPTLVTTLTYGV
QCFSRYPDHMKQHDFFKSAMPEGYVQERTIFFKDDGNYKTRAEVKFEGDTLVNRIELKGIDFKEDGNILGHKL
EYNYNSHNVYIMADKQKNGIKVNFKIRHNIEDGSVQLADHYQQNTPIGDGPVLLPDNHYLSTQSALSKDPNEKR
DHMVLLEFVTAAGITLGMDELYKSGGSPKKKRKV

Protein Sequence of CBEΔUGI-YE1:

MKRTADGSEFESPKKKRKVSSETGPVAVDPTLRRRIEPHEFEVFFDPRELRKETCLLYEINWGGRHSIWRHTS
QNTNKHVEVNFIEKFTTERYFCPNTRCSITWFLSYSPCGECSRAITEFLSRYPHVTLFIYIARLYHHADPENRQG
LRDLISSGVTIQIMTEQESGYCWRNFVNYSPSNEAHWPRYPHLWVRLYVLELYCIILGLPPCLNILRRKQPQLTF
FTIALQSCHYQRLPPHILWATGLKSGGSSGGSSGSETPGTSESATPESSGGSSGGSDKKYSIGLAIGTNSVGW
AVITDEYKVPSKKFKVLGNTDRHSIKKNLIGALLFDSGETAEATRLKRTARRRYTRRKNRICYLQEIFSNEMAKV
DDSFFHRLEESFLVEEDKKHERHPIFGNIVDEVAYHEKYPTIYHLRKKLVDSTDKADLRLIYLALAHMIKFRGHFLI
EGDLNPDNSDVDKLFIQLVQTYNQLFEENPINASGVDAKAILSARLSKSRRLENLIAQLPGEKKNGLFGNLIALSL
GLTPNFKSNFDLAEDAKLQLSKDTYDDDLDNLLAQIGDQYADLFLAAKNLSDAILLSDILRVNTEITKAPLSASMI
KRYDEHHQDLTLLKALVRQQLPEKYKEIFFDQSKNGYAGYIDGGASQEEFYKFIKPILEKMDGTEELLVKLNRE
DLLRKQRTFDNGSIPHQIHLGELHAILRRQEDFYPFLKDNREKIEKILTFRIPYYVGPLARGNSRFAWMTRKSEE
TITPWNFEEVVDKGASAQSFIERMTNFDKNLPNEKVLPKHSLLYEYFTVYNELTKVKYVTEGMRKPAFLSGEQK
KAIVDLLFKTNRKVTVKQLKEDYFKKIECFDSVEISGVEDRFNASLGTYHDLLKIIKDKDFLDNEENEDILEDIVLTL
TLFEDREMIEERLKTYAHLFDDKVMKQLKRRRYTGWGRLSRKLINGIRDKQSGKTILDFLKSDGFANRNFMQLI
HDDSLTFKEDIQKAQVSGQGDSLHEHIANLAGSPAIKKGILQTVKVVDELVKVMGRHKPENIVIEMARENQTTQ
KGQKNSRERMKRIEEGIKELGSQILKEHPVENTQLQNEKLYLYYLQNGRDMYVDQELDINRLSDYDVDHIVPQS
FLKDDSIDNKVLTRSDKNRGKSDNVPSEEVVKKMKNYWRQLLNAKLITQRKFDNLTKAERGGLSELDKAGFIK
RQLVETRQITKHVAQILDSRMNTKYDENDKLIREVKVITLKSKLVSDFRKDFQFYKVREINNYHHAHDAYLNAVV
GTALIKKYPKLESEFVYGDYKVYDVRKMIAKSEQEIGKATAKYFFYSNIMNFFKTEITLANGEIRKRPLIETNGET
GEIVWDKGRDFATVRKVLSMPQVNIVKKTEVQTGGFSKESILPKRNSDKLIARKKDWDPKKYGGFDSPTVAYS
VLVVAKVEKGKSKKLKSVKELLGITIMERSSFEKNPIDFLEAKGYKEVKKDLIIKLPKYSLFELENGRKRMLASAG
ELQKGNELALPSKYVNFLYLASHYEKLKGSPEDNEQKQLFVEQHKHYLDEIIEQISEFSKRVILADANLDKVLSA
YNKHRDKPIREQAENIIHLFTLTNLGAPAAFKYFDTTIDRKRYTSTKEVLDATLIHQSITGLYETRIDLSQLGGDKR
TADGSEFEPKKKRKVGSGATNFSLLKQAGDVEENPGPMVSKGEELFTGVVPILVELDGDVNGHKFSVSGEGE
GDATYGKLTLKFICTTGKLPVPWPTLVTTLTYGVQCFSRYPDHMKQHDFFKSAMPEGYVQERTIFFKDDGNYK
TRAEVKFEGDTLVNRIELKGIDFKEDGNILGHKLEYNYNSHNVYIMADKQKNGIKVNFKIRHNIEDGSVQLADHY
QQNTPIGDGPVLLPDNHYLSTQSALSKDPNEKRDHMVLLEFVTAAGITLGMDELYKSGGSPKKKRKV

Protein Sequence of dABE-F148A:

MKRTADGSEFESPKKKRKVSEVEFSHEYWMRHALTLAKRAWDEREVPVGAVLVHNNRVIGEGWNRPIGRHD
PTAHAEIMALRQGGLVMQNYRLIDATLYVTLEPCVMCAGAMIHSRIGRVVFGARDAKTGAAGSLMDVLHHPGM
NHRVEITEGILADECAALLSDAFRMRRQEIKAQKKAQSSTDSGGSSGGSSGSETPGTSESATPESSGGSSGG
SSEVEFSHEYWMRHALTLAKRARDEREVPVGAVLVLNNRVIGEGWNRAIGLHDPTAHAAIMALRQGGLVMQN
YRLIDATLYVTFEPCVMCAGAMIHSRIGRVVFGVRNAKTGAAGSLMDVLHYPGMNHRVEITEGILADECAALLC
YAFRMPRQVFNAQKKAQSSTDSGGSSGGSSGSETPGTSESATPESSGGSSGGSDKKYSIGLAIGTNSVGWA
VITDEYKVPSKKFKVLGNTDRHSIKKNLIGALLFDSGETAEATRLKRTARRRYTRRKNRICYLQEIFSNEMAKVD
DSFFHRLEESFLVEEDKKHERHPIFGNIVDEVAYHEKYPTIYHLRKKLVDSTDKADLRLIYLALAHMIKFRGHFLIE
GDLNPDNSDVDKLFIQLVQTYNQLFEENPINASGVDAKAILSARLSKSRRLENLIAQLPGEKKNGLFGNLIALSLG
LTPNFKSNFDLAEDAKLQLSKDTYDDDLDNLLAQIGDQYADLFLAAKNLSDAILLSDILRVNTEITKAPLSASMIKR
YDEHHQDLTLLKALVRQQLPEKYKEIFFDQSKNGYAGYIDGGASQEEFYKFIKPILEKMDGTEELLVKLNREDLL
RKQRTFDNGSIPHQIHLGELHAILRRQEDFYPFLKDNREKIEKILTFRIPYYVGPLARGNSRFAWMTRKSEETITP
WNFEEVVDKGASAQSFIERMTNFDKNLPNEKVLPKHSLLYEYFTVYNELTKVKYVTEGMRKPAFLSGEQKKAI
VDLLFKTNRKVTVKQLKEDYFKKIECFDSVEISGVEDRFNASLGTYHDLLKIIKDKDFLDNEENEDILEDIVLTLTL
FEDREMIEERLKTYAHLFDDKVMKQLKRRRYTGWGRLSRKLINGIRDKQSGKTILDFLKSDGFANRNFMQLIHD
DSLTFKEDIQKAQVSGQGDSLHEHIANLAGSPAIKKGILQTVKVVDELVKVMGRHKPENIVIEMARENQTTQKG
QKNSRERMKRIEEGIKELGSQILKEHPVENTQLQNEKLYLYYLQNGRDMYVDQELDINRLSDYDVDHIVPQSFL
KDDSIDNKVLTRSDKNRGKSDNVPSEEVVKKMKNYWRQLLNAKLITQRKFDNLTKAERGGLSELDKAGFIKRQ

LVETRQITKHVAQILDSRMNTKYDENDKLIREVKVITLKSKLVSDFRKDFQFYKVREINNYHHAHDAYLNAVVGT
ALIKKYPKLESEFVYGDYKVYDVRKMIAKSEQEIGKATAKYFFYSNIMNFFKTEITLANGEIRKRPLIETNGETGEI
VWDKGRDFATVRKVLSMPQVNIVKKTEVQTGGFSKESILPKRNSDKLIARKKDWDPKKYGGFDSPTVAYSVLV
VAKVEKGKSKKLKSVKELLGITIMERSSFEKNPIDFLEAKGYKEVKKDLIIKLPKYSLFELENGRKRMLASAGELQ
KGNELALPSKYVNFLYLASHYEKLKGSPEDNEQKQLFVEQHKHYLDEIIEQISEFSKRVILADANLDKVLSAYNK
HRDKPIREQAENIIHLFTLTNLGAPAAFKYFDTTIDRKRYTSTKEVLDATLIHQSITGLYETRIDLSQLGGDSGGSK
RTADGSEFEPKKKRKVGSGATNFSLLKQAGDVEENPGPMVSKGEELFTGVVPILVELDGDVNGHKFSVSGEG
EGDATYGKLTLKFICTTGKLPVPWPTLVTTLTYGVQCFSRYPDHMKQHDFFKSAMPEGYVQERTIFFKDDGNY
KTRAEVKFEGDTLVNRIELKGIDFKEDGNILGHKLEYNYNSHNVYIMADKQKNGIKVNFKIRHNIEDGSVQLADH
YQQNTPIGDGPVLLPDNHYLSTQSALSKDPNEKRDHMVLLEFVTAAGITLGMDELYKSGGSPKKKRKV

Protein Sequence of dCBE-YE1:

MKRTADGSEFESPKKKRKVSSETGPVAVDPTLRRRIEPHEFEVFFDPRELRKETCLLYEINWGGRHSIWRHTS
QNTNKHVAVNFIEKFTTERYFCPNTRCSITWFLSYSPCGECSRAITEFLSRYPHVTLFIYIARLYHHADPENRQG
LRDLISSGVTIQIMTEQESGYCWRNFVNYSPSNEAHWPRYPHLWVRLYVLELYCIILGLPPCLNILRRKQPQLTF
FTIALQSCHYQRLPPHILWATGLKSGGSSGGSSGSETPGTSESATPESSGGSSGGSDKKYSIGLAIGTNSVGW
AVITDEYKVPSKKFKVLGNTDRHSIKKNLIGALLFDSGETAEATRLKRTARRRYTRRKNRICYLQEIFSNEMAKV
DDSFFHRLEESFLVEEDKKHERHPIFGNIVDEVAYHEKYPTIYHLRKKLVDSTDKADLRLIYLALAHMIKFRGHFLI
EGDLNPDNSDVDKLFIQLVQTYNQLFEENPINASGVDAKAILSARLSKSRRLENLIAQLPGEKKNGLFGNLIALSL
GLTPNFKSNFDLAEDAKLQLSKDTYDDDLDNLLAQIGDQYADLFLAAKNLSDAILLSDILRVNTEITKAPLSASMI
KRYDEHHQDLTLLKALVRQQLPEKYKEIFFDQSKNGYAGYIDGGASQEEFYKFIKPILEKMDGTEELLVKLNRE
DLLRKQRTFDNGSIPHQIHLGELHAILRRQEDFYPFLKDNREKIEKILTFRIPYYVGPLARGNSRFAWMTRKSEE
TITPWNFEEVVDKGASAQSFIERMTNFDKNLPNEKVLPKHSLLYEYFTVYNELTKVKYVTEGMRKPAFLSGEQK
KAIVDLLFKTNRKVTVKQLKEDYFKKIECFDSVEISGVEDRFNASLGTYHDLLKIIKDKDFLDNEENEDILEDIVLTL
TLFEDREMIEERLKTYAHLFDDKVMKQLKRRRYTGWGRLSRKLINGIRDKQSGKTILDFLKSDGFANRNFMQLI
HDDSLTFKEDIQKAQVSGQGDSLHEHIANLAGSPAIKKGILQTVKVVDELVKVMGRHKPENIVIEMARENQTTQ
KGQKNSRERMKRIEEGIKELGSQILKEHPVENTQLQNEKLYLYYLQNGRDMYVDQELDINRLSDYDVDHIVPQS
FLKDDSIDNKVLTRSDKNRGKSDNVPSEEVVKKMKNYWRQLLNAKLITQRKFDNLTKAERGGLSELDKAGFIK
RQLVETRQITKHVAQILDSRMNTKYDENDKLIREVKVITLKSKLVSDFRKDFQFYKVREINNYHHAHDAYLNAVV
GTALIKKYPKLESEFVYGDYKVYDVRKMIAKSEQEIGKATAKYFFYSNIMNFFKTEITLANGEIRKRPLIETNGET
GEIVWDKGRDFATVRKVLSMPQVNIVKKTEVQTGGFSKESILPKRNSDKLIARKKDWDPKKYGGFDSPTVAYS
VLVVAKVEKGKSKKLKSVKELLGITIMERSSFEKNPIDFLEAKGYKEVKKDLIIKLPKYSLFELENGRKRMLASAG
ELQKGNELALPSKYVNFLYLASHYEKLKGSPEDNEQKQLFVEQHKHYLDEIIEQISEFSKRVILADANLDKVLSA
YNKHRDKPIREQAENIIHLFTLTNLGAPAAFKYFDTTIDRKRYTSTKEVLDATLIHQSITGLYETRIDLSQLGGDSG
GSGGSGGSTNLSDIIEKETGKQLVIQESILMLPEEVEEVIGNKPESDILVHTAYDESTDENVMLLTSDAPEYKPW
ALVIQDSNGENKIKMLSGGSGGSGGSTNLSDIIEKETGKQLVIQESILMLPEEVEEVIGNKPESDILVHTAYDEST
DENVMLLTSDAPEYKPWALVIQDSNGENKIKMLSGGSKRTADGSEFEPKKKRKVGSGATNFSLLKQAGDVEE
NPGPMVSKGEELFTGVVPILVELDGDVNGHKFSVSGEGEGDATYGKLTLKFICTTGKLPVPWPTLVTTLTYGV
QCFSRYPDHMKQHDFFKSAMPEGYVQERTIFFKDDGNYKTRAEVKFEGDTLVNRIELKGIDFKEDGNILGHKL
EYNYNSHNVYIMADKQKNGIKVNFKIRHNIEDGSVQLADHYQQNTPIGDGPVLLPDNHYLSTQSALSKDPNEKR
DHMVLLEFVTAAGITLGMDELYKSGGSPKKKRKV

Protein Sequence of dCBEΔUGI-YE1:

MKRTADGSEFESPKKKRKVSSETGPVAVDPTLRRRIEPHEFEVFFDPRELRKETCLLYEINWGGRHSIWRHTS
QNTNKHVAVNFIEKFTTERYFCPNTRCSITWFLSYSPCGECSRAITEFLSRYPHVTLFIYIARLYHHADPENRQG
LRDLISSGVTIQIMTEQESGYCWRNFVNYSPSNEAHWPRYPHLWVRLYVLELYCIILGLPPCLNILRRKQPQLTF
FTIALQSCHYQRLPPHILWATGLKSGGSSGGSSGSETPGTSESATPESSGGSSGGSDKKYSIGLAIGTNSVGW
AVITDEYKVPSKKFKVLGNTDRHSIKKNLIGALLFDSGETAEATRLKRTARRRYTRRKNRICYLQEIFSNEMAKV
DDSFFHRLEESFLVEEDKKHERHPIFGNIVDEVAYHEKYPTIYHLRKKLVDSTDKADLRLIYLALAHMIKFRGHFLI
EGDLNPDNSDVDKLFIQLVQTYNQLFEENPINASGVDAKAILSARLSKSRRLENLIAQLPGEKKNGLFGNLIALSL
GLTPNFKSNFDLAEDAKLQLSKDTYDDDLDNLLAQIGDQYADLFLAAKNLSDAILLSDILRVNTEITKAPLSASMI

KRYDEHHQDLTLLKALVRQQLPEKYKEIFFDQSKNGYAGYIDGGASQEEFYKFIKPILEKMDGTEELLVKLNRE
DLLRKQRTFDNGSIPHQIHLGELHAILRRQEDFYPFLKDNREKIEKILTFRIPYYVGPLARGNSRFAWMTRKSEE
TITPWNFEEVVDKGASAQSFIERMTNFDKNLPNEKVLPKHSLLYEYFTVYNELTKVKYVTEGMRKPAFLSGEQK
KAIVDLLFKTNRKVTVKQLKEDYFKKIECFDSVEISGVEDRFNASLGTYHDLLKIIKDKDFLDNEENEDILEDIVLTL
TLFEDREMIEERLKTYAHLFDDKVMKQLKRRRYTGWGRLSRKLINGIRDKQSGKTILDFLKSDGFANRNFMQLI
HDDSLTFKEDIQKAQVSGQGDSLHEHIANLAGSPAIKKGILQTVKVVDELVKVMGRHKPENIVIEMARENQTTQ
KGQKNSRERMKRIEEGIKELGSQILKEHPVENTQLQNEKLYLYYLQNGRDMYVDQELDINRLSDYDVDHIVPQS
FLKDDSIDNKVLTRSDKNRGKSDNVPSEEVVKKMKNYWRQLLNAKLITQRKFDNLTKAERGGLSELDKAGFIK
RQLVETRQITKHVAQILDSRMNTKYDENDKLIREVKVITLKSKLVSDFRKDFQFYKVREINNYHHAHDAYLNAVV
GTALIKKYPKLESEFVYGDYKVYDVRKMIAKSEQEIGKATAKYFFYSNIMNFFKTEITLANGEIRKRPLIETNGET
GEIVWDKGRDFATVRKVLSMPQVNIVKKTEVQTGGFSKESILPKRNSDKLIARKKDWDPKKYGGFDSPTVAYS
VLVVAKVEKGKSKKLKSVKELLGITIMERSSFEKNPIDFLEAKGYKEVKKDLIIKLPKYSLFELENGRKRMLASAG
ELQKGNELALPSKYVNFLYLASHYEKLKGSPEDNEQKQLFVEQHKHYLDEIIEQISEFSKRVILADANLDKVLSA
YNKHRDKPIREQAENIIHLFTLTNLGAPAAFKYFDTTIDRKRYTSTKEVLDATLIHQSITGLYETRIDLSQLGGDKR
TADGSEFEPKKKRKVGSGATNFSLLKQAGDVEENPGPMVSKGEELFTGVVPILVELDGDVNGHKFSVSGEGE
GDATYGKLTLKFICTTGKLPVPWPTLVTTLTYGVQCFSRYPDHMKQHDFFKSAMPEGYVQERTIFFKDDGNYK
TRAEVKFEGDTLVNRIELKGIDFKEDGNILGHKLEYNYNSHNVYIMADKQKNGIKVNFKIRHNIEDGSVQLADHY
QQNTPIGDGPVLLPDNHYLSTQSALSKDPNEKRDHMVLLEFVTAAGITLGMDELYKSGGSPKKKRKV

TadA

TadA*

rAPOBEC1

Cas9

UGI

EGFP

SV40 NLS

Supplementary Tables

Supplementary Table 1. List of DNA repair genes categorized by DNA repair pathways (in separate excel file)

Supplementary Table 2. Protospacer and PAM sequences for all sites used for assessing editing efficiencies.

	Protospacer	PAM
HEK2	GAAC_4AC_6AAAGCATAGACTGC	GGG
HEK3	GGC$C_4$$C_5$AGACTGAGCACGTGA	TGG
RNF2	GTC_3ATC_6TTAGTCATTACCTG	AGG
HIRA	GAAGA_5CCAAGGATAGACTGC	TGG
PSMB2	GTAAA_5CA_7AAGCATAGACTGA	GGG
HEK4	GGCAC_5TGCGGCTGGAGGTGG	GGG
HL1	ATTC_4TACCAGAGGTACAAGG	AGG

Supplementary Table 3. First round genomic DNA PCR primer sequences

Primer Name	Primer Sequence
HEK2-Fwd	ACACTCTTTCCCTACACGACGCTCTTCCGATCTNNNNATTGTCCAGCCCCATCTGTCAA
HEK2 -Rev	TGGAGTTCAGACGTGTGCTCTTCCGATCTTTCAAGTTACTGCAGCCCAAGC
HEK3-Fwd	ACACTCTTTCCCTACACGACGCTCTTCCGATCTNNNNGAGACAGGGATCCCAGGGAAAC
HEK3-Rev	TGGAGTTCAGACGTGTGCTCTTCCGATCTCCCAGCCAAACTTGTCAACCAG
RNF2-Fwd	ACACTCTTTCCCTACACGACGCTCTTCCGATCTNNNNGCAGACAAACGGAACTCAACCA
RNF2-Rev	TGGAGTTCAGACGTGTGCTCTTCCGATCTCCCACCACTGTTCACCCCAGTACCT
HIRA-Fwd	ACACTCTTTCCCTACACGACGCTCTTCCGATCTNNNNGCATCATAGCGAGACCCTGTCT
HIRA-Rev	TGGAGTTCAGACGTGTGCTCTTCCGATCTTTTGGCCCAATGACACCACATG
PSMB2 (pos 7)-Fwd	ACACTCTTTCCCTACACGACGCTCTTCCGATCTNNNNACTGTGACTGGCCCCCAATATC

PSMB2 (pos 7)-Rev	TGGAGTTCAGACGTGTGCTCTTCCGATCTTACCCCTGTTCCTAAAGCCCAC
HEK4-Fwd	CCTCCCTTCAAGATGGCTGACANNNNACTGTGACTGGCCCCCAATATC
HEK4-Rev	TGGAGTTCAGACGTGTGCTCTTCCGATCTCCCAGTGTCTCCGTTCGGGTTGAAAG
HL1-Fwd	AAGAGTCCAGGACCAGATGGATNNNNACTGTGACTGGCCCCCAATATC
HL1-Rev	TGGAGTTCAGACGTGTGCTCTTCCGATCTCCCACATTCTGATACCAAAGCCGGG
